# Determinants of Sweetness Preference: A Scoping Review of Human Studies

**DOI:** 10.3390/nu12030718

**Published:** 2020-03-08

**Authors:** Carolina Venditti, Kathy Musa-Veloso, Han Youl Lee, Theresa Poon, Alastair Mak, Maryse Darch, Justine Juana, Dylan Fronda, Daniel Noori, Erika Pateman, Maia Jack

**Affiliations:** 1 Intertek Health Sciences, Inc., Suite 201, 2233 Argentia Road, Mississauga, ON L5N 2X7, Canada; 2 American Beverage Association, Science and Regulatory Affairs, 1275 Pennsylvania Ave. NW, Washington, DC 200042, USA

**Keywords:** sweet, sweetness, liking, preference

## Abstract

Factors associated with sweetness preference are multi-faceted and incredibly complex. A scoping review was undertaken to identify determinants of sweetness preference in humans. Using an online search tool, ProQuest ™, a total of 99 publications were identified and subsequently grouped into the following categories of determinants: Age, dietary factors, reproductive hormonal factors, body weight status, heritable, weight loss, sound, personality, ethnicity and lifestyle, previous exposure, disease, and ‘other’ determinants. Methodologies amongst studies were heterogenous in nature (e.g., there was variability across studies in the sweetness concentrations tested, the number of different sweetness concentrations used to assess sweetness preference, and the methods utilized to measure sweetness preference), rendering interpretation of overall findings challenging; however, for certain determinants, the evidence appeared to support predictive capacity of greater sweetness preference, such as age during certain life-stages (i.e., young and old), being in a hungry versus satiated state, and heritable factors (e.g., similar sweetness preferences amongst family members). Recommendations for the design of future studies on sweetness preference determinants are provided herein, including an “investigator checklist” of criteria to consider.

## 1. Introduction

Human preference for sweet foods is universal, with hedonic responses changing over a person’s lifetime [1]. Sweet molecules in nature are sugars found primarily in plants (i.e., fructose, sucrose, and glucose), in addition to lactose found in many species’ milk, all of which provide a source of energy and sweetness. It has been hypothesized that sweetness preference may exist to identify energy-rich foods (i.e., containing readily available glucose) [2], which provides necessary metabolic fuel for the brain [3]. However, with the advent of non-nutritive sweeteners (NNS), debate as to how these are influencing sweetness preference has gained momentum within the scientific community. The signaling of sweet taste is a complex system involving a ligand (a sweet molecule) binding to a receptor, which activates downstream signaling to indicate either the perception of taste or the modulation of further signaling pathways.

Taste receptors are proteins that recognize one of the five taste modalities—salty, sweet, bitter, sour, and umami (a savory sensation)—and facilitate the sensation of taste [4]. The detection of “sweetness” occurs mainly through a G-coupled protein receptor, i.e., a heterodimeric plasma membrane receptor composed of a taste 1 receptor member 2 (T1R2) and taste 1 receptor member 3 (T1R3) [5,6]. Their associated genes are TAS1R2 and TAS1R3, respectively [7]. It has previously been suggested that T1R3, alone, can function as a low-affinity taste receptor for sugar (but not NNS) [7,8]. Polymorphisms may lead to differences between individuals in sweetness preferences. Processing of sweet tastes occurs first within the oral cavity, where receptors (i.e., T1R2+T1R3 and T1R3 alone) are clustered in taste buds on the tongue to detect sweet flavors. The activation of the receptors results in cell depolarization and neurotransmitter secretion to communicate sweetness to the brain [9].

Interestingly, sweet taste receptors have been identified beyond the oral cavity, including but not limited to the visual, auditory and olfactory systems [10,11,12], the gastrointestinal (GI) tract, the pancreas [13,14], and the respiratory tract, including the nose and lungs [15] where “sweetness” itself is not perceived, but receptor activation may lead to downstream effects [13]. Additionally, a role for sweet taste receptors has been suggested in the initiation and/or progression of some pathological processes and metabolic disease, such as asthma (i.e., in the respiratory tract) or diabetes (i.e., in the pancreas) [14,16]. In the GI tract, sweet taste receptors are thought to play a supportive role in glucose absorption and metabolism [6], as well as the release of satiety hormones, such as glucagon-like peptide-1 [9]. Overall, regardless of the location in the body, there appear to be many downstream mechanisms that may occur following the activation of sweet taste receptors [2].

**Figure 1:**
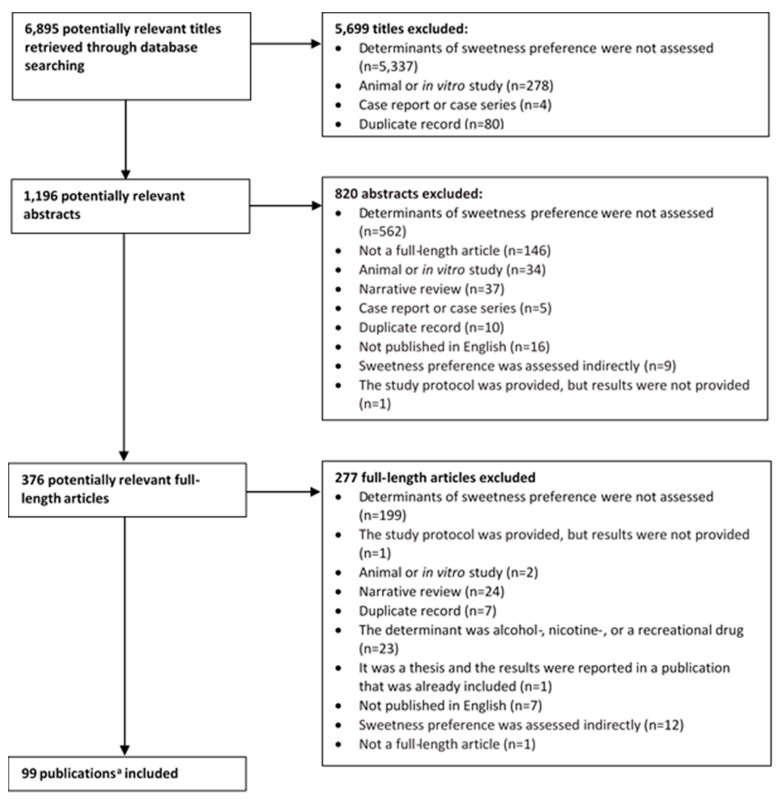
Flowchart of the literature search process. ^a^ Two of the publications [23,24] were considered “kin studies” (i.e., the studies were conducted using the same population of individuals, and outcomes were assessed at the same time point, but different outcomes were published in different manuscripts). Therefore, 98 unique studies (99 publications) are included.

The location and interaction of sweet taste receptors in the body are incredibly complex (reviewed by Lee et al. [4], Fernstrom et al. [9], Welcome et al. [17]). The effects of ligands binding to the sweet taste receptors are pleiotropic, and the location of the receptor in the body often determines its functionality. Sweet taste receptors have an affinity for multiple sweet-tasting substrates. Although caloric sugars (e.g., glucose, lactose, fructose, galactose, sucrose, and maltose), NNS (e.g., aspartame, sucralose, acesulfame potassium, saccharin, steviol glycosides), and sweet proteins (e.g., monellin, thaumatin, and brazzein) all have very different chemical structures, these sweet-tasting substrates all interact with the same taste receptor (T1R2+T1R3), yet on different regions of the receptor (see Figure 1 in the publications by Fernstrom et al. [9] and Sanematsu et al. [18]). When the sweet-taste antagonist, lactisole, binds the sweet taste receptors, the perception of all sweet taste is inhibited (reviewed by Beauchamp [2], Jiang et al. [19]). Some NNS also bind bitter taste receptors (T2R). For example, saccharin binds to the T2R bitter taste receptor in addition to the sweet receptor and accounts for the “bitter aftertaste” often attributed to its consumption [9].

While it has been suggested that preference for sweetness is nature’s way of allowing humans to identify foods containing readily available glucose or energy [2], in actuality, the degree of a food’s sweetness provides little information about its total energy value [20,21]. Ramirez [21] proposed that the primary function of sweet preference is the cue for sweetness. It is possible that sweetness may act as a signal for ripeness of foods (e.g., fruits), and thereby the presence of easily metabolizable sugars (e.g., the provision of very rapid glucose sources, unlike the case for fats and protein) [22]. It should be noted that an individual’s gauge for sweetness preference is separate (but not necessarily mutually exclusive) from sweetness sensitivity and detection. However, the determinants of a person’s preference for sweetness are largely unknown. Evidence published in this domain is diverse and provides many different hypotheses regarding influencing factors leading to an individual’s preferences for sweetness. Sweetness preference is a complex, multi-faceted topic, and corresponding determinants are likely numerous; a scoping review was therefore undertaken to understand the breadth of such a large and varied evidence-base. In an attempt to summarize the data, the following research question was formulated: “Based on published scientific literature, what is the current knowledge-base regarding the many different determinants of a person’s preference for sweetness and what are the key research focus areas?”

## 2. Materials and Methods

### 2.1. Literature Search Strategy

The objective of this scoping review was to map the available scientific evidence on sweetness preference determinants in humans. To retrieve relevant literature, the electronic search tool, ProQuest Dialog ™, was used to search four literature databases: BIOSIS Previews ^®^, CAB ABSTRACTS, Embase ^®^, and MEDLINE ^®^. Two different literature searches were conducted. In the first literature search, three sets of keywords were searched [i.e., keywords for the exposure (i.e., substances that impart a sweet taste), keywords for sweetness preference, and keywords for study population]. For a study to be identified, at least one keyword from each of the three keyword categories had to appear either in the title or abstract of the article. In the second literature search, only articles in which the terms “effect” or “effects” preceded the terms “sweetness” or “sweet taste”—and these terms were separated by not more than 15 terms in the article titles or abstracts (e.g., “effects of gender on sweetness preference...”)—would be identified. The keywords that were used in the first and second literature searches can be found in Table S1-1 of Supplementary File S1. To increase search relevance, articles that were not original, peer-reviewed publications (e.g., conference abstracts, editorial, letter, reviews) were excluded. The literature searches were initially conducted on 28 November 2017 and were subsequently updated on 1 October 2018.

#### 2.1.1. Inclusion and Exclusion Criteria

Publication eligibility was assessed at the title, abstract, and full text levels. A study was included if it met all of the following eligibility criteria: (1) A human study; (2) an intervention or observational study in which sweetness preference determinants were investigated; (3) as a measure of sweetness preference, subjects had to rate their preference (e.g., by rating their preference on a scale, ranking test samples in order of preference) or there was a direct assessment of the intake of foods that served as a proxy for sweetness preference (e.g., there was a direct assessment of ad libitum orange juice consumption), or a questionnaire related to the desire for sweetness was administered (studies in which food frequency questionnaires, diaries, or records of specific “sweet foods” consumed in the past were not considered eligible for inclusion); (4) alcohol, nicotine, or a recreational drug relative to sweetness preference determinants were considered out-of-scope; (5) a full-length primary research study published in a peer-reviewed journal (e.g., not review); and (6) published in English.

#### 2.1.2. Data Extraction, Study Assessment, and Synthesis of Results

Studies were screened and assessed by five reviewers; each reviewer was proficient at literature assessment and sorting, based on previous experience. Each reviewer was provided with a set of titles to sort through based on a predetermined set of inclusion/exclusion criteria (as described above); abstracts of potentially relevant titles were then reviewed, and finally, full-text articles of potentially relevant abstracts were reviewed for eligibility. Where systematic reviews were identified, referenced studies within, that met all of the inclusion criteria for this scoping review, were also included as part of the evidence-base. Studies that met all the inclusion criteria were categorized into groups with similar determinants. In some studies, multiple determinants were investigated. For the purposes of this scoping review, the outcome most relevant to the primary objective of the particular study served as the basis for its categorization. A study characteristics table was developed to provide a template for relevant data extraction from each of the studies identified for the scoping review. The following data were extracted: (1) Author; (2) year of publication; (3) study population (including the number of subjects, gender distribution, and whether subjects were children or adults); (4) sweetness test delivery matrix; (5) type of sweetener; (6) levels of sweetness tested; (7) how sweetness preference was assessed; and (8) top-level results relevant to the relationship between the determinant investigated and sweetness preference. The data were then compiled independently by two reviewers (each reviewer had a specific set of included publications from which to extract relevant information for the table), and the results were verified by a third researcher. Inconsistencies were discussed amongst reviewers. Unresolved discrepancies were settled by a third party. Illustrations summarizing extracted data were created in an attempt to convey the breadth and depth of key determinants and contextualizing information such as vehicles delivering sweetening agents and corresponding sweetness levels.

### 2.2. Additional Analyses

Once pertinent publications (meeting all eligibility criteria) were identified and the results tabulated, it became evident that the designs of the experimental studies (defined as those studies in which sweetness preference was assessed by presenting the subjects with one or more sweet solutions or foods and having them rate their likeness of the solution[s]/food[s]) were highly heterogeneous. A checklist of methodological items, “Considerations in the Design of Sweetness Preference Studies: An Investigator Checklist”, (“investigator checklist”) was prepared by the authors of this paper and included factors that may improve future study designs assessing sweetness preference and elements (and accompanying justification) within the following four categories: (1) Standardization of procedures for examiners; (2) standardization of subject characteristics; (3) standardization of procedures for subjects; and (4) standardization of sweetness preference testing (see Table 1 and Table S2-1 of Supplementary File S2).

The “investigator checklist” was applied to the experimental studies identified in this scoping review. For each checklist item, a study received a score of one (1) if that parameter was reported on in the study or zero (0) otherwise. If the study objective was to determine a given parameter’s association with sweetness preference (e.g., whether body weight status was associated with sweetness preference), then that parameter was rated as not applicable (NA) in the “investigator checklist”; this was because this variable would not have been controlled in the study population, but rather would have been the predictor variable under investigation. After the two reviewers completed the “investigator checklist” for their respective pool of studies, each reviewer randomly selected and reviewed a quarter of the studies assessed by the other reviewer to ensure consistency in scoring. Where disagreements persisted, a third reviewer was tasked with identifying a resolution. Challenges experienced during the scoring of the studies were utilized to provide further clarification on the rating of the said parameter (i.e., listed under the column heading “Additional considerations applied to the scoring parameter” in Table S2-1 of Supplementary File S2). Results were tallied, and trends and/or inconsistencies were reported.

## 3. Results

### 3.1. Literature Search Results

As depicted in the flowchart in Figure 1, the literature searches yielded 6895 titles from which 1196 abstracts were retrieved, with 376 potentially relevant full-length articles. Ultimately, 99 publications were relevant for the scoping review. Of the 99 full-length publications, two of the publications [23,24] were considered “kin studies” (i.e., the studies were conducted using the same population of individuals, and outcomes were assessed at the same time point, but different outcomes were published on in the different manuscripts). The publications by Drewnowski et al. [23,24] were therefore included as one study, resulting in 98 unique studies, yet 99 publications.

### 3.2. Categorization of Studies According to the Sweetness Preference Determinant Investigated

**Figure 2:**
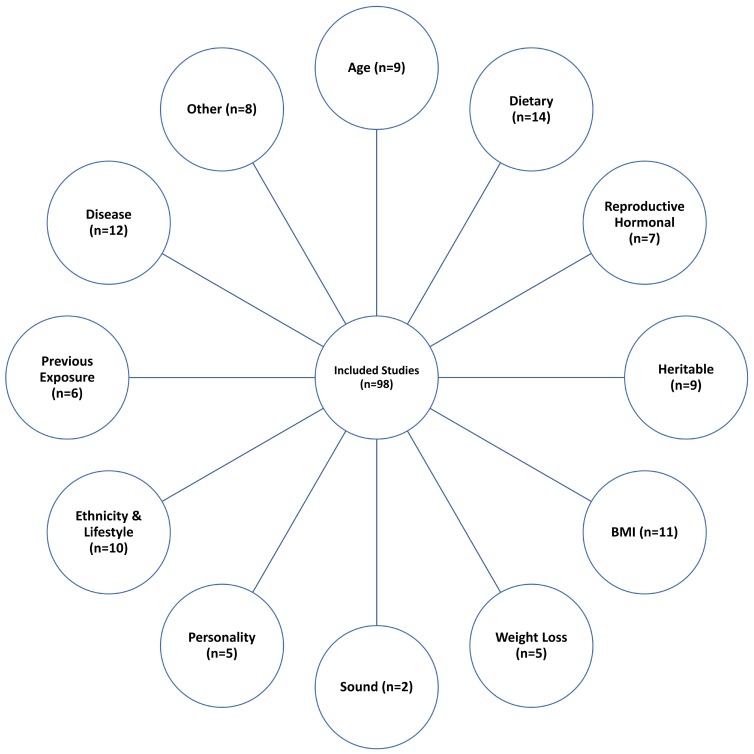
Sweetness preference distinct determinants.

There were 12 distinct categories of sweet taste determinants (Figure 2): Disease (*n* = 12 studies), age (*n* = 9 studies), dietary factors (*n* = 14 studies), reproductive hormonal factors (*n* = 7 studies), body weight status/body mass index (BMI) (*n* = 11 studies), heritable/genetic influences (*n* = 9 studies), weight loss (*n* = 5 studies), sound (*n* = 2 studies), personality traits (*n* = 5 studies), ethnicity and lifestyle (*n* = 10 studies), previous exposure (*n* = 6 studies), and ‘other’ determinants (*n* = 8 studies).

### 3.3. Key Study Characteristics

In assessing the determinants of sweetness preference, the following were considered to be key study characteristics: Sweetener used to assess sweetness preference, physical state (solid, semi-solid, or liquid) of the vehicle used to deliver the sweetener, sweetness concentrations tested (as sucrose equivalents), the number of different sweetness concentrations used to assess sweetness preference, and methods utilized to measure sweetness preference (i.e., subject’s rating, ranking, choice, intake of foods, or response to questionnaires).

In 90 of the 98 studies, sweetness preference was assessed experimentally by having the study participant sample at least one set of sweetened foods or beverages. A “set of sweetened foods or beverages” was defined as one or more variations of the same food or beverage, tested in the same study population, sweetened with the same sweetener (i.e., sugar or NNS), but differing only in the level of sweetness. In the remaining eight studies [25,26,27,28,29,30,31,32], sweetness preference was assessed without having subjects sample and rate a sweetened food or beverage. For example, Mizuta et al. [26] requested study participants with different leptin and leptin gene polymorphisms to score from 1 (“I hate them”) to 5 (“I love them”) when asked, “Do you like things that taste sweet?” Importantly, however, self-reported data were different from sensory or hedonic data. A subset analysis to exclude these self-reports would likely not change the results, as they represented a small fraction of all studies for any given parameter.

**Figure 3:**
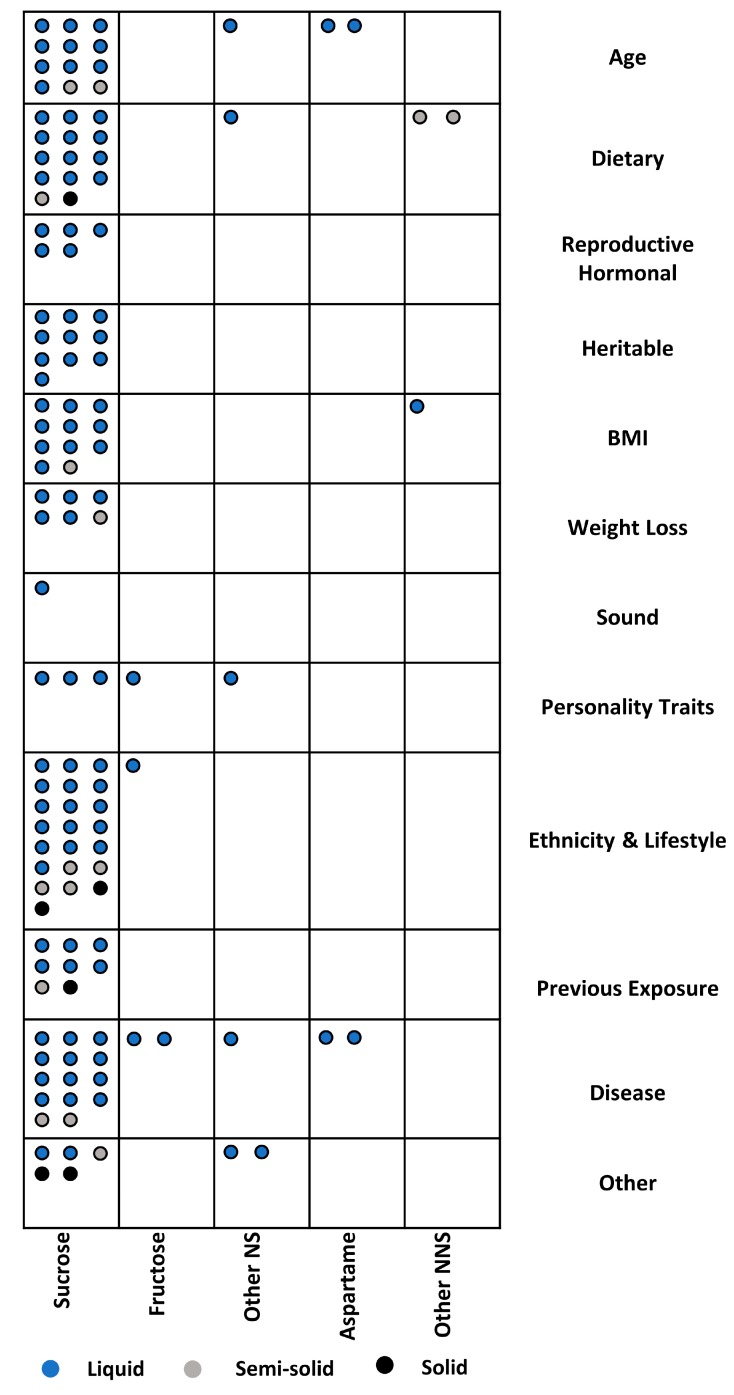
Sweeteners and physical states of sweetness delivery vehicles in sweetness reference studies. Each circle represents a different food/beverage set within the 98 studies evaluated, where a “set” is defined as one or more variations of the same food or beverage, tested in the same study population, sweetened with the same sweetener (i.e., NS or NNS), but differing only in the level of sweetness. There are a total of 128 food/beverage sets. In several studies, assessments of sweetness preference were completed for multiple “sets”. NS = nutritive sweetener; NNS = non-nutritive sweetener.

**Figure 4:**
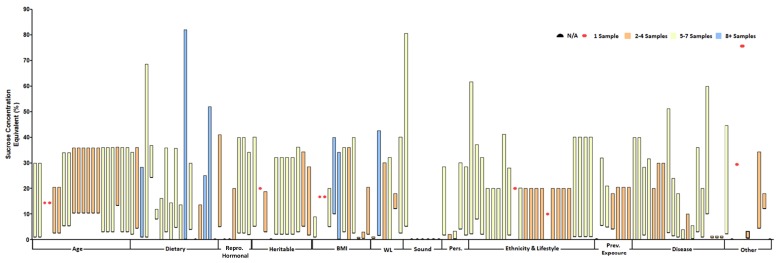
Sweetness levels (sucrose concentration equivalents) tested across studies. Each study’s reported sweetness level (i.e., a single sweet food/beverage set tested) or range (i.e., more than one sweetness level assessed for a food/beverage set) is represented by vertical bars, as sucrose equivalents, for each distinct determinant.

A total of 128 sets of sweetened foods or beverages were tested across the 90 studies in which sweetness preference was assessed experimentally (Figure 3). In most sets (i.e., 111), sucrose was used as the sweetener. Fructose or another nutritive sweetener (NS) was used in 10 sets. Aspartame or another NNS was used in only seven sets. Additionally, the majority (i.e., 107) were in liquid form, with only six and 15 in solid or semi-solid form, respectively. Moreover, the sweetener concentration (to achieve a certain level of sweetness) in the test foods/beverages was provided for 126 sets (Figure 4). The sucrose concentration exceeded 40% (wt/vol or wt/wt) in 13 sets. In just over half the sets (i.e., 51%), five to seven sweetness levels were tested. In nine sets, only one level of sweetness was used to determine sweetness preference.

### 3.4. Study Results

#### 3.4.1. Age and Sweetness Preference

In nine studies, the effect of age on sweetness preference was assessed (Table S3-1 of Supplementary File S3). In four of the experimental studies, the sweetness preferences of children or adolescents were compared to those of young adults [33,34,35,36]. Children and adolescents tended to have a significantly greater sweetness preference compared to young adults. One study showed that children had a significantly greater sweetness preference than adolescents [35]. In another study, elderly individuals had a significantly greater preference for sucrose-sweetened iced tea than young adults; however, there was a significant age-by-gender effect when individuals consumed iced tea sweetened with aspartame. For example, elderly males preferred sweeter iced tea than did elderly females and young males, but not compared with young females [37]. In the remaining four studies, children had a significantly greater sweetness preference than their mothers, independent of the sweetening agent (sucrose, sucralose, or aspartame) [38,39,40,41]. Importantly, Bobowski and Mennella [41] observed significant differences in sweetness preference between children and their mothers only when using a five-point facial hedonic scale, but not when using a three-point facial hedonic scale. Thus, overall, there appears to be a U- or J-shaped curve characterizing sweetness preference, such that sweetness preference is high in childhood, decreases with advancing age, but then increases in elderly, perhaps more so in elderly males than females.

#### 3.4.2. Dietary/Nutritional Factors and Sweetness Preference

In 14 studies, the effects of dietary/nutritional factors on sweetness preference were assessed (Table S3-2 of Supplementary File S3). In six of these studies, the effects of a fasted versus fed state on sweetness preference were evaluated [42,43,44,45,46,47]. Only Pangborn [42] reported no significant differences in sweetness preference among participants segregated based on the self-reported level of hunger (consumer study) or based on a fed versus fasted state (laboratory study). It is not clear whether the test beverage (i.e., sucrose-sweetened peach-flavored drink or apricot-flavored nectars) influenced the outcome, as most studies in this scoping review tested sweetness preference with sucrose-sweetened water solutions. Martin et al. [47] reported an increased sweetness preference with increased hunger, irrespective of gender. Laeng et al. [46] also observed a significantly higher sweetness preference, but only among females in a ‘hungry’ state compared to their satiated ‘fed’ counterparts. Looy and Weingarten [45] observed a significant increase in sweetness preference in sweet dislikers when hungry versus satiated. However, in sweet likers, there was no significant difference in sweetness preference in the satiated versus hungry state. Moskowitz et al. [43] observed that three (i.e., hungry males or satiated males who consumed either breakfast or lunch) of the four groups studied demonstrated a liking of glucose solutions up to 1 M, beyond which there was a reduction in liking; however, in the fourth group, which included males who had been given an oral glucose load, glucose solutions in excess of 1 M were still enjoyed. In contrast, Fantino et al. [44] reported statistically significant reductions in female sweetness preference following an oral glucose load. Thus, in most instances, a fasted state resulted in an increase in sweetness preference, though there were often other situation-specific findings, such as gender effects or effects of sweetness liking.

In the remaining eight studies, sweetness preference was investigated as a function of several different nutritional or dietary variables. Zhou et al. [31] reported a significantly greater desire for sweetness among females (but not males) after consuming two lunch test meals (low in vegetables and carbohydrates, yet high in meat), but not after consuming four other lunch test meals varying in fat, vegetable, and meat content. Tatano et al. [43] reported a significant increase in sweetness preference among adults who were habitual consumers of a high-fat diet (i.e., ≥25% of total energy) compared to those on a lower-fat diet (i.e., <25% of total energy). Wise et al. [48] reported no significant differences in sweetness preference among adults consuming a habitual diet or a low sugar diet, using either sucrose-sweetened vanilla pudding or sucrose-sweetened raspberry beverage as the test vehicle. When adults were segregated based on degree of sweet intake (i.e., low, medium, or high intake), significant differences were observed across all three groups, with high (sweet) intake consumers having a significantly greater sweetness preference than medium (sweet) intake consumers, who in turn had a significantly greater sweetness preference than low (sweet) intake consumers [49]. Similarly, Garneau et al. [50] reported significantly greater intakes of sweetened juice and sweetened tea among sweet likers and sweet neutrals compared to sweet dislikers, even though there were no significant differences in water or total energy intakes [50]. In one of two cross-sectional studies in children, candy and snack consumption (but not sweet drink, cereal, dairy, fruit product, or added sugar consumption), was significantly associated with sweetness preference [51]. Similarly, significant positive correlations were observed between sweetness preference and the number of snack occasions and sweet intake occasions [52], yet the opposite was observed between sweetness preference and the number of main meals consumed. Vazquez et al. [53] found no significant differences between well-nourished and malnourished infants, suggesting similar sweetness preferences between these infant groups.

Overall, there was no clear pattern for sweetness preference based on dietary macronutrient composition or meal composition; however, there does appear to be some consistency in the literature with regard to a general increase in sweetness preference in the fasted versus satiated state, though there were notable study-specific findings (e.g., effects of gender, consumption of glucose preloads, general sweetness liking, etc.).

#### 3.4.3. Reproductive Hormonal Factors and Sweetness Preference

In seven studies, the effects of feminine hormonal factors on sweetness preference were assessed (Table S3-3 of Supplementary File S3). In one study, which included adolescents at various stages of puberty, no significant differences between pubertal stage and sweetness preference were identified [54]. In two studies, the effects of oral contraceptive use on sweetness preference were assessed. In one study, there were no significant differences in the amounts of sweet foods consumed (i.e., jelly babies, milk chocolate, or digestive cookies) in users compared to non-users of oral contraceptives [27], while in a different study, women taking low-progestin compared to high-progestin oral contraceptives had a significantly greater liking for sweet solutions [55]. In another study, while no significant differences in sweetness preference were observed during the three pregnancy trimesters, pregnant women compared to non-pregnant women had a significantly lower sweetness preference [55]. In the four remaining studies, differences in women’s sweetness preference throughout the various phases of the menstrual cycle were assessed [56,57,58,59]. Brown and Grunfeld [58] reported an increase in the amount of sweet foods (i.e., cake, chocolate, gumdrops) consumed by women during the luteal versus follicular phase of their menstrual cycle. Frye et al. [59] reported an increase in sweetness preference (over a 4-week period) when study initiation coincided with the women’s luteal or menstrual phase of the menstrual cycle. However, Wright and Crow [56] observed a significant reduction in sweetness preference during the luteal phase relative to the other menstrual phases. Additionally, at 10 min and 1 h following the consumption of a glucose load, sweetness preference decreased significantly in women at all phases of the menstrual cycle, except for women in the ovulation phase, where a decrease was not observed at 10 min after glucose load consumption [56]. Likewise, Pliner and Fleming [57] reported a significant reduction in sweetness preference 15 or 30 min after the ingestion of a glucose load when women were in the luteal phase but not the follicular phase of their menstrual cycles.

In one study, sweetness preference was not associated with stage of puberty in adolescents; and, in another study, pregnant women were noted to have a decreased sweetness preference than non-pregnant women. Without additional studies, it is not possible to assess the consistency of these associations. The associations between sweetness preferences and the different menstrual cycle phases were inconsistent.

#### 3.4.4. Genetics and Sweetness Preference

In nine studies, the effects of genetics on sweetness preference were assessed (Table S3-4 of Supplementary File S3). In two of these studies, the concordance in sweetness preference was assessed between monozygotic or dizygotic twins, either in children [60] or in female adults [61]. Heritability explained a significant proportion of the variability in sweetness preference. In a third study, which was conducted in 146 male and female adults from 26 Finnish families, it was reported that heritability was significantly associated with preference for the two sweetest sucrose solutions tested [62]. In another study, it was reported that leptin gene A19G and leptin receptor gene R109K polymorphisms were significantly associated with having a sweet tooth [26]. In the five remaining studies, sweetness preference was assessed as a function of sensitivity to the bitter-tasting 6-n-propylthiouracil (PROP). Sensitivity to PROP is a heritable trait, whereby persons are divided into non-, regular (medium), or supertasters of PROP; most supertasters appear to be women, with increased taste buds anatomically [63]. Some studies have reported that increased sensitivity to PROP is associated with the increased sweet perception of sucrose and saccharin at low concentrations [64,65]; but the results are inconsistent, at best [66]. In three of the five studies, all of which were conducted in females, there were no significant associations between PROP sensitivity and sweetness preference [23,24,67,68]. In contrast, in a study in male and female adults by Yeomans et al. [69], PROP supertasters had a significantly reduced preference for the sweetest sucrose solutions compared with PROP non- and medium tasters. Only in the study by Mennella et al. [70] was sensitivity to PROP determined genetically (as opposed to phenotypically). In this study, which included children and their mothers, being heterozygous or homozygous for the bitter-sensitive allele was associated with a statistically significant increased sweetness preference than being homozygous for the bitter insensitive allele in the children but not in the mothers. Limited evidence hints at some hereditary influence on sweetness preference.

#### 3.4.5. Body Weight and Sweetness Preference

In 11 studies—eight in adults and three in children—associations between body weight and sweetness preference were assessed (Table S3-5 of Supplementary File S3). Of the eight studies conducted in adults, four were conducted solely in females [71,72,73,74], and in three of these studies, there were no significant differences between normal weight and overweight/obese females in their sweetness preference [71,72,73]. However, Connolly et al. [74] found that obese women had a significantly lower preference for Ocean Spray Cranberry juice sweetened either with Truvia or sucrose compared to normal weight women. Of note, in this study, both diet and caloric beverages were used, but only at a single strength/concentration, unlike the other studies in which there were four to 10 solutions tested that varied in their sweetness levels. In the other four adult studies, which included both females and males [75,76,77,78], there were no significant differences between the normal weight and overweight/obese groups in their sweetness preference; in three of these studies, sweetness preference was assessed using sucrose solutions, while in the study by Wooley et al. [75], sweetness preference was assessed before and 1 h after ingestion of 200 mL of a 25% glucose solution or 200 mL of an isosweet cyclamate solution, on two different study days.

Of the three studies conducted in children, two showed no significant differences between normal weight and overweight/obese children [79] or between infants of non-obese and infants of obese mothers in their sweetness preference [80]. Though the findings in each study were similar, it is interesting to note that both the vehicle and the concentration of sweetness differed; apple juice, 0.4% and 1.0% sucrose wt/vol in the study conducted by Alexy et al. [79] and water, 2.0% to 20.5% sucrose wt/vol in the study conducted by Grinker et al. [80]. While in the third study the vehicle (apple juice) and concentrations (0.53% and 3.11 sucrose % wt/vol) for sweetness were similar to those studied by Alexy et al. [79], children who preferred sweeter apple juice tended to be overweight or obese [81]. However, total energy intakes were not included in the multivariate model. The collective evidence suggests there is no effect on sweetness preference based on weight status across age groups and across genders.

#### 3.4.6. Weight Loss and Sweetness Preference

In five experimental studies, the effects of weight loss on sweet preference were assessed (Table S3-6 of Supplementary File S3). In three of these studies, sweetness preference was assessed both before and after weight loss [82,83,84]. In two studies, it was reported that sweetness preference was not affected by weight loss, neither in obese children, obese adolescents [82], or obese adults [83], following a 12- and 6-month weight loss intervention, respectively. In contrast, in the study by Burgess et al. [84], obese females reported a significantly greater preference for only the strawberry milk sweetened with 15% sucrose following a 6-month weight loss program, whereas before the weight loss program there was a significant preference for strawberry milk sweetened with 15% or 30% sucrose over 0% sucrose. The latter findings suggest that the weight loss intervention among these obese females reduced their sweetness preference for the 30% sucrose solution. In the remaining two studies, both of which were conducted only in females, while a weight loss intervention was not included as part of the study, sweetness preference was assessed as a function of the degree of weight fluctuations—high or low—[85] or recent or past weight loss [86]. Compared with women with a low degree of weight fluctuations, women with a high degree of weight fluctuations were found to have a significantly greater sweet taste preference for sucrose when added to ice cream. However, the preference for sucrose-sweetened water was the same in women with a high versus a low degree of weight fluctuations [85]. Women who had lost a large amount of weight in the past had a significantly lower sweetness preference than the women who had lost a small amount of weight in the past, both before and following an oral glucose load. In contrast, women with a recent weight loss of a “high” quantity, had a significantly greater sweetness preference than women with a recent weight loss of a “low” quantity, following (but not prior to) a glucose load [86]. More studies are needed to assess the effects of weight loss and weight fluctuations on sweetness preferences.

#### 3.4.7. Sound and Sweetness Preference

In two studies, the effects of sound on sweetness preference were assessed (Table S3-7 of Supplementary File S3). Ferber and Cabanac [87] reported that, compared to silence, loud noise and music were associated with a significantly greater sense of pleasure for all the sucrose stimuli. Kontukoski et al. [29] reported that listening to “sweet” versus “sour” music resulted in a significant increase in the use of self-selected sweet ingredients during the preparation of beverages. With only two studies assessing the effects of sound on sweet preference, it is not possible to draw conclusions on the consistency of the results.

#### 3.4.8. Personality Traits and Sweetness Preference

In five studies, different personality traits and their associations with sweetness preference were assessed (Table S3-8 of Supplementary File S3). Kampov-Polevov et al. [88] reported an association between sweetness preference (of the strongest sucrose solution) and either mood or uncontrolled eating habits. Sweetness preference was associated with many different personality traits, including having greater impulsiveness or lower openness [89], being a novice alcohol-drinker or less extroverted [90], having greater choice impulsivity [91], and being highly outgoing or having a low or medium type A personality [92]. Together, the limited evidence is highly heterogeneous when attempting to assess the effects of various personality traits on sweetness preference, with no clear or consistent associations.

#### 3.4.9. Ethnicity and Lifestyle and Sweetness Preference

Sweetness preference was assessed as a function of ethnicity or lifestyle in 10 studies (Table S3-9 of Supplementary File S3). Specifically, sweetness preferences were assessed among: (1) Different ethnic/cultural groups [93,94,95,96,97]; (2) individuals with traditional versus modern lifestyles [98,99,100]; and (3) athletes versus less active individuals [101,102].

For the different cultural/ethnic groups evaluated, two studies were conducted in children [94,96] and three in adults [93,95,97]. Takemi and Woo [96] reported no significant differences between Korean and Japanese children in their sweetness preference when offered banana milks sweetened with different sucrose levels. Jaafar and Abdul Razak [94] found that Malay and Indian children had a significantly greater sweetness preference of different sucrose solutions than did Chinese children, all of whom were living in Malaysia. Tuorila et al. [97] reported no significant differences in the sweetness preference of a single 20% sucrose solution (wt/vol), between British and Finnish adults. Bertino et al. [93] compared the sweetness preferences of university students who were studying in the United States (U.S.), including European descent U.S.-born students, and Chinese descent Taiwan-born students, and found test food used played a significant role. Specifically, when sucrose solutions were used in the hedonic testing, there was a near-statistically significant increased sweetness preference in the students of Chinese descent relative to those of European descent (*p* = 0.06); in contrast, when cookies sweetened with different levels of sucrose were used, there was a statistically significant sucrose-by-group interaction, such that the students of Chinese descent rated the least sweet cookies as tasting most pleasant. A similar observation between sweetness preference and test food was made by Holt et al. [95] when they compared sweet preferences of Australian university students, who were either born in Australia (and were Caucasian) or born in Malaysia, [and were primarily (73%) of Malay descent]. In Australians (compared to Malaysian-descent), the mean sucrose rating was significantly greater when consuming shortbread biscuits, but was significantly lower when consuming orange juice or custard. When sucrose solutions were used as the test food, Malaysians had a significantly greater preference for the 8% sucrose solution and a significantly lower preference for the 32% solution relative to Australians. Furthermore, a statistically significant correlation was observed between the most preferred sucrose solution and the frequency of consumption of sweet foods and drinks and total and refined sugars. Collectively, the evidence does suggest cultural and ethnic influences on sweetness preference, with westernized cultures (i.e., Americans, Europeans, Australians) preferring both more highly sweetened foods and beverages compared to their Asian counterparts. The spectrum for sweetness preference is likewise greater among South Asian cultures (i.e., Malaysian, Indian) compared to mid-Asian cultures (i.e., Chinese, Japanese, Korean). However, the concept of increased liking due to the familiarization of foods (rather than to sweetness preference) cannot be ruled out.

In all three studies in which sweetness preference was assessed as a function of traditional versus modern lifestyle [98,99,100], a modern lifestyle was associated with a significantly greater sweetness preference than a traditional lifestyle. Specifically, white adults were observed to have a significantly greater sweetness preference than Pima Indians [99], and individuals living in urban areas in Iraq were observed to have a significantly greater sweet taste preference than individuals living in rural areas in Iraq [98]. Additionally, Sorokowska et al. [100] reported that Poles (a modern society) and Tsimane’ (forager horticulturalists) had a significantly greater sweetness preference than the Hadza (hunter-gatherers living in Tanzania). Here again, the evidence suggests that a modern lifestyle likely influences sweetness preference when comparing to traditional dietary patterns. A modern lifestyle may provide an increased opportunity for food familiarization, lending itself to an increased perception of sweetness preference. Sweetness sensitivity must be considered when interpreting the results of these studies.

In the two studies in which the sweetness preferences of individuals with different physical activity levels were assessed, the more physically active the individual, the lower their sweetness preference [101,102]; thus, there is some suggestion, albeit from a very limited number of studies, that increased physical activity may be associated with a reduction in sweetness preference.

#### 3.4.10. Previous Sweetness Exposure and Sweetness Preference

The effects of early-life exposure to sweetness on sweetness preference later in life were assessed in six studies (Table S3-10 of Supplementary File S3). Specifically, the effects of exposure to sweetened water during early infancy on subsequent sweetness preference were assessed in two studies [103,104]. Additionally, the effects of previous exposure to sweet foods on subsequent sweetness preference in infants or children were assessed in four studies [58,105,106,107].

Exposure to sweetened water during infancy was associated with a significantly greater intake of sweetened solutions at 6 months [103] and at 2 years [104], compared with children who were not administered sweetened solutions during infancy. Interestingly, however, Beauchamp and Moran [104] observed that when an unfamiliar beverage was used as the test food (i.e., Kool-Aid), intakes were not significantly different between 2-year-old children who had and had not consumed sweetened water as infants. When the children were then segregated according to prior exposure to Kool-Aid, those who had previously consumed Kool-Aid had a significantly greater intake of Kool-Aid than those who had not previously consumed Kool-Aid.

Fry Vennerød et al. [107] reported that children with more frequent exposures to sweet and snack foods had a significantly greater sweetness preference than children who had fewer exposures to sweets and snacks. In contrast, Liem et al. [105] reported that children whose parents highly restricted their intakes of sugars, versus those whose parents did not, had the highest sweetness preference. On the other hand, the consumption of baby foods with added sugars during the first 3 months of life had no association with the liking of sweetened foods during the fourth month of life [58]. Similarly, young children’s sweetness preference was not associated with the type of formula they consumed as infants (i.e., milk-based versus hydrolysate). When children were grouped according to those whose mothers routinely added sugar to their foods, those whose mothers consumed added sugars had a significantly greater sweetness preference than those whose mothers did not consume added sugars [105].

Overall, it appears that previous exposure and possibly familiarity (versus unfamiliarity) of sweetened foods/beverages may be associated with increased sweetness preference of similar foods/beverages at later timepoints. That sweetness preference was increased in children whose parents highly restricted sugar intakes suggest that sweetness preference is not just a function of previous exposure and familiarity.

#### 3.4.11. Disease and Sweetness Preference

Twelve studies were identified wherein the effects of disease on sweetness preference were investigated (Table S3-11 of Supplementary File S3). Seven of the studies included subjects with a neurological or psychological disease [108,109,110,111,112,113,114], three included subjects with type 2 diabetes mellitus (T2DM) or gestational diabetes mellitus (GDM) [115,116,117], and two included subjects with other diseases, namely end-stage renal disease or Prader-Willi Syndrome (PWS) [118,119]. All studies but one were conducted in adults. The study in patients with end-stage renal disease was conducted in children [118].

##### Studies in Subjects with a Neurological or Psychological Disease

In patients with Parkinson’s disease (PD), while there was no overall difference in sucrose preference when compared to control subjects, statistically significant differences were observed at higher sucrose concentrations (and therefore increased sweetness) (*p* = 0.003) and for the interaction between disease state and sucrose concentration (*p* < 0.001) [109]. In another study, no differences were observed [112]. Patients suffering from depression showed no differences in their sweetness preference when compared with individuals who were not suffering from depression [111,113]. In anorexic restrictors, there was a significantly lower preference for the least sweet sucrose solutions when compared to healthy controls and anorexic bulimics. Similarly, when sucrose concentrations were provided in a dairy beverage, there was a significantly lower preference for the non-sweetened dairy beverage in anorexic restrictors compared to controls and anorexic bulimics [108]. Franko et al. [110] reported a significantly greater preference for the sweetest sucrose solution in patients with narrowly defined bulimia nervosa compared with healthy controls and adults with bulimia nervosa plus a history of anorexia nervosa [114] found that adults with binge-eating disorders fell into two categories, namely higher sweet preferences and lower sweet preferences; those in the higher sweet preference category had a significantly greater frequency of binge-eating and over-eating (i.e., as measured in the previous 28 days of the study) relative to those in the lower sweet preference category.

##### Studies in Individuals with T2DM or GDM

Tepper et al. [116] found that individuals with or without T2DM had similar sweetness preferences when consuming a cherry-flavored beverage sweetened with either sucrose, fructose, or aspartame, while Yu et al. [117] found that individuals with T2DM exhibited a significantly lower sweetness preference. In contrast, Tepper and Seldner [115] found that pregnant women with GDM (compared to their non-GDM counterparts) had a significantly greater preference for the sweetest strawberry-flavored milk (sweetened with sucrose), but no significant difference was observed in their preference for a glucose-sweetened solution.

##### Studies in Patients with Other Diseases

Children with end-stage renal disease compared to healthy children had a significantly lower sweetness preference, irrespective of the food delivery matrix (i.e., soft white cheese or apple sauce) [118]. Compared to healthy individuals, individuals with PWS had a significantly greater sweetness preference, irrespective of the sweetener used (i.e., sucrose, fructose, or aspartame) [119]. No clear pattern emerges relative to disease state and sweetness preferences. Differences observed depend on the specific disease and experimental conditions associated with the study.

#### 3.4.12. Other Factors and Sweetness Preference

Other factors investigated as sweetness preference determinants were found in eight studies (Table S3-12 of Supplementary File S3). Frijters [120] reported no significant differences in sweetness preference in females who were low-, medium-, or high-restrained eaters. Weight-stable women who had Roux-en-Y gastric bypass and who thereafter received leptin had a significant reduction in cravings for sweets than did women who received a placebo [28]. Stressed females were found to consume significantly more sweet, high fat, and high fat sweet foods than females who were not stressed [121]. In a study of prospective nutrition teachers, no significant associations were observed between sweetness preference and several variables, including physical activity level, stress level, sleep, weight gain, or constipation [122]. In four cross-sectional studies, significant positive correlations were identified between sweetness preference and several different variables, including sweet taste sensitivity, fasting blood glucose level, basal metabolic rate, body weight index [123]; total energy and carbohydrate (total sugar, fructose, glucose) intakes [124]; country of residence, age, and BMI [125]; and age, current smoking status, stronger cognitive restraint, and former dieting ([30]; refer to Table S3-12 of Supplementary File S3 for gender-specific results). Lampuré et al. [30] found significant negative correlations between sweetness preference and uncontrolled eating habits and emotional eating habits (see Table S3-12 of Supplementary File S3 for gender-specific results).

The above investigations exemplify the level of interest in evaluating the complexities associated with sweet preference and corresponding determinants. The available evidence is incredibly diverse.

#### 3.4.13. Summary of Findings

A summary of the overall findings, as reported in Section 3.4.1, Section 3.4.2, Section 3.4.3, Section 3.4.4, Section 3.4.5, Section 3.4.6, Section 3.4.7, Section 3.4.8, Section 3.4.9, Section 3.4.10, Section 3.4.11 and Section 3.4.12, is provided in Table 2.

#### 3.4.14. Findings related to the “investigator checklist”

**Figure 5:**
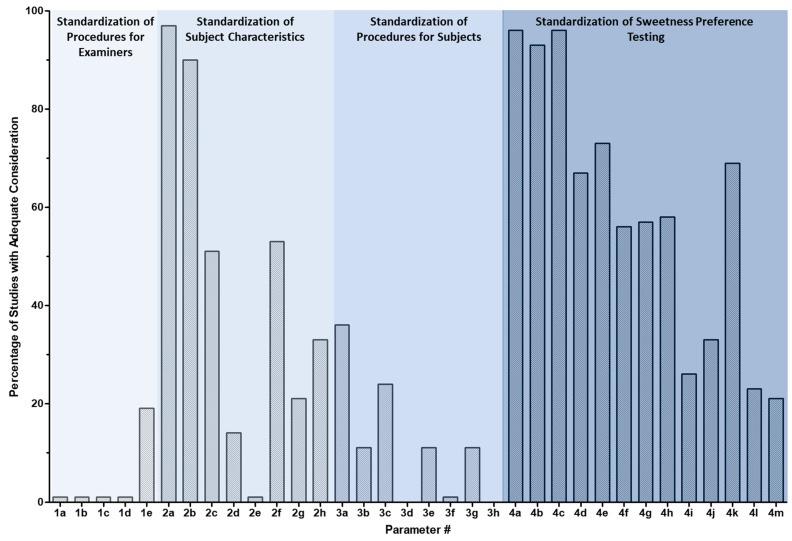
Summary of the scoring parameters from the “investigator checklist”. The percentage of studies in which adequate consideration of the identified parameter in the investigator checklist is provided. The “investigator checklist” is available in Table S2-1 of Supplementary File S2; an abbreviated version of the checklist is available in Table 1.

The “investigator checklist” was developed to assess the methodological robustness of the included studies and is intended to provide guidance in the development of future clinical study protocols for studies assessing determinants of sweetness preference. It was based on the reporting of the studies included in this scoping review; great variability in study design—and a lack of reporting of confounding factors and covariates—was evident. The “investigator checklist” was applied to the 90 experimental studies identified in this scoping review (a copy of the scoring for each study is provided in Supplementary File S4). For the remaining eight studies, sweetness preference was not assessed using sweetness taste testing; therefore, the “investigator checklist” was not applicable for these studies. For all but one of the 90 studies, four of the five examiner’s characteristics (i.e., Items 1a to 1d of the “investigator checklist”, relating to smoking, use of scented personal products, consumption of strong-smelling confectionary, or avoidance of consumption of foods with strong odors, respectively) were not at all standardized or controlled. The criteria that were most commonly addressed in the studies included age (i.e., Parameter 2a) (96% of studies) and gender (i.e., Parameter 2b) (91% of studies), sweetener tested (i.e., Parameter 4a) (94% of studies), sweetness concentration tested (i.e., Parameter 4b) (91% of studies), and vehicle composition (i.e., Parameter 4c) (92% of studies). In less than 37% of the studies were confounding factors and covariates in the hours leading up to sweetness testing well-controlled (i.e., Section 3, Parameters 3a to 3g of the “investigator checklist”, including subject fasting, last meal consumed, use of tobacco products, alcohol consumption, personal use of scented products, consumption of strongly scented confectionary, and physical activity, respectively). Amongst the 90 studies, the minimum and maximum percentages of items accounted for ranged from 13% to 57%, with 37% being the mean proportion of items accounted for. A summary of the scores for the parameters on the “investigator checklist” is provided in Figure 5.

## 4. Discussion

In this scoping review, a mapping of the evidence related to sweetness preference determinants was undertaken and shows how complex the study of sweetness preference is. The determinants were grouped into 12 distinct categories, including age, dietary, reproductive hormonal factors, heritable/genetic, body weight status, weight loss, sound, personality, ethnicity and lifestyle, previous exposure, disease, or other. Study designs were heterogeneous, and findings largely inconsistent across studies, rendering interpretation challenging, at best. In the experimental studies identified herein, sucrose was the most commonly used sweetener and water was the most commonly used vehicle to deliver the sucrose, while the other testing parameters—including the range in sweetness tested, the number of sweetness concentrations tested, and the methodologies used to assess sweetness preference—were highly variable. In a recent review conducted by Iatridi et al. [126], the authors indicated that amongst the 71 studies wherein sweet taste likers were assessed, a lack of alignment existed between the studies in regard to the methods used to assess sweetness preference. Furthermore, the authors indicated that “…a better understanding of individual variations in sweet taste perception could clarify how sweet-liking interplays with obesity … The development of a universally used statistically robust and less time-consuming classification method is needed” [126]. This conclusion is consistent with that of the scoping review presented herein, namely that there is a need for a standardized approach in the study of sweetness preference among the general population.

A summary of the overall findings of this scoping review is presented in Table 2. From the available evidence, a number of consistent findings emerged despite the heterogeneity in experimental designs, some of which are further described below. Inconsistent findings were distilled and summarized in Table 2 only.

Overall, age (more than any other factor) was associated with sweetness preference. Children, followed by adolescents (i.e., ≤10 years old, 11–18 years old, respectively) tended to have the highest preference for sweet-tasting foods [33,34,35,36,81]. It has been suggested that a child’s preference for sweetness is an evolutionary (and innate) trait intended to increase energy consumption, necessary for this critical life-stage [1,127]. Some have suggested in utero exposure to sweet taste in amniotic fluid may likewise affect later acceptance of foods [16], while others have suggested maternal routine consumption of added sugars may increase sweetness preference in their children versus those of mothers who did not [105]. Interestingly, sweetness preference also increases among elderly individuals (i.e., >60 years of age) [37], which may be explained in part by decreases in taste perception due to reduced taste sensitivity and taste discrimination [128,129,130,131]. Age-related changes in sweetness preference among the elderly do not appear to be due to physical changes in the number of taste buds in the gustatory system [132]—but rather due to alterations in brain function and/or structure over time (e.g., reduced grey matter or cortical thickness) [133,134,135].

Heritability appeared to influence sweetness preference [39,40,60,61,62]. Although one’s preference for sweet-tasting foods is likely partially due to environmental factors and/or conditions during a child’s upbringing [136], nearly 50% may be mediated through heritable genes, as assessed in identical versus fraternal twins [60,61] and likely localized to a genetic marker on chromosome 16 [62]. Additional research has shown that genetic variation in the *TAS1R3* sweet receptor gene leads to differences in sweetness preference in adults, but not children [40,49].

Most studies did not show an association between body weight status and sweetness preference [71,72,73,76,77,78,79,80]. In one study, a significantly lower preference for sweetness among obese women compared to their normal weight counterparts was reported [74]. Collectively, these important findings are relevant and suggest that overconsumption of foods in obese/overweight individuals may not necessarily be due to sweetness preference but rather due broadly to food preference [137,138].

Findings evaluating the association between prior exposure to sweetened foods with possible subsequent sweetness preference are inconsistent, likely due to differences in study duration and study test materials and confounders due to food familiarity [58,102,103]. While several studies did not find an association between prior exposure and subsequent sweetness preference, two studies reported that children with, as opposed to without, more frequent exposures to sweet and snack foods had significantly greater sweetness preferences [105,107]. However, conditioning and familiarization relative to prior sweetness exposure and later sweetness preference may be confounded by the other, rendering interpretation of these types of analyses challenging [93,94,95,96,97,98,99,100,139,140,141,142]. While limited evidence suggests prior exposure to sweetened foods may impact sweetness preference in subsequent exposures, future studies should attempt to more clearly address food familiarization as a possible confounder.

There were mixed results regarding reproductive hormonal effects on one’s sweetness preference. Coldwell et al. [54] indicated that puberty in male and female adolescents (i.e., significant hormonal changes) did not appear to affect one’s preference for sweetness. It should be noted that the authors did not control for the menstrual cycle in females. An increase in progesterone levels (i.e., a key hormone in both pregnancy and the menstrual cycle [143] during the luteal phase of the menstrual cycle) was associated with an increase in food consumption [58] and an increase in sweetness preference [59]. Low-dose, but not high-dose, progestin (i.e., a form of progesterone) oral contraceptives also led to a significant increase in sweetness preference [55]. In pregnant women, no effect on sweetness liking was observed throughout gestation, although pregnant women had a significantly lower preference for sweetness than women who were not pregnant [55]. Perhaps an inverse relationship exists between progesterone levels and sweetness preference where high levels of progesterone (such as those in pregnancy) lead to a decrease in preference for sweet foods, but low levels of progesterone (e.g., luteal phase of menstrual cycle or low-dose progestin oral contraceptives) lead to an increase in sweetness preference. Although Faas et al. [144] suggested an increase in progesterone throughout pregnancy appeared to increase a woman’s food intake, sweet preference per se was not discussed. Additional studies evaluating sweetness preference in accordance with specific levels of hormones, including progesterone, should be conducted.

Among the included studies, a considerable amount of variability existed, which may have contributed to the inconsistent findings among many of the studies/determinants. In some studies, the vehicle used to assess sweetness preference—e.g., sucrose solutions compared to sucrose added to a food/beverage—was shown to independently influence the hedonic response to sweetness [93,95,115]. Assessing sweetness preference with anything other than sweetened water solutions would likely introduce the confounding effect from the food matrix per se. Solutions that vary only in their sweetness (as opposed to foods and beverages that vary in macronutrient composition—the latter possibly impacting palatability as well as sweetness perception and preference) are likely the more appropriate test vehicles for evaluating sweet preference.

Likewise, the range of sweetness could explain inconsistent findings between studies. Some studies suggest a “breakpoint” concentration at about 1 M or 35% glucose (wt/vol) may exist, such that beyond this level, sweet preference declines among individuals due to test solutions being perceived as overly sweet [43]. Using test concentrations beyond this “breakpoint” could facilitate the identification of sweet “likers” from “dislikers”. For instance, Travers et al. [109] noted a significant disease-by-concentration effect among individuals with PD, such that liking increased with increased sucrose concentration; yet, Sienkiewicz-Jarosz et al. [112] noted no significant differences in sweetness preference between patients with versus without PD, likely explained by the limited range of sweetness tested—i.e., no more than 30% sucrose solution compared to 51.3% in the study by Travers et al. [109]. Similarly, discordant results were reported between studies evaluating sweetness preferences among: (1) Adults with (compared to without) T2DM [116,117]; and (2) obese or overweight (compared to non-obese) children [79,81]. Null findings were reported in those studies that used less-concentrated sucrose-sweetened test beverages [79,116]. Future studies should ensure the range of sweetness tested exceeds 35% (wt/vol) or 1 M glucose.

In some studies, the use of a single test food/beverage with only one sweetness level proved limiting, as it may have precluded the subjects’ ability to discern sweetness preference and/or to evaluate the concept of dose-response [e.g., Tuorila et al. [97] reported no significant differences between Finnish and British adults in their hedonic rating of a 20% sucrose solution]. Additional doses of the test food/beverage may have enabled the determination of a difference in sweetness preference between groups. Furthermore, a limited number of studies evaluated sweeteners other than sucrose, and preliminary findings suggest that sweetness preference may be dependent on the sweetener used. For example, sweetness preference differed between the consumption of iced tea sweetened with sucrose versus aspartame [37].

Finally, existing assessment tools that evaluate sweet preference may not be adequate. Tucci et al. [27] relied on amounts of sweet foods consumed (i.e., jelly babies, milk chocolate, or digestive cookies) to assess the degree of sweet preference, which unfortunately could be confounded not only by the food matrix per se but also by the familiarity of the food. Bobowski and Mennella [41] found that, by using a five-point rather than a three-point hedonic scale for sweetness preference, differences between children and their mothers were statistically significant. This study highlights the need for more granularity in assessment tools to discern differences in sweetness preference between population groups.

Limitations of the scoping review included: (1) Sorting (i.e., against inclusion/exclusion criteria) of the potentially relevant titles identified was not assessed in duplicate due to the sheer volume of titles (approximately 7000) that had to be filtered through (rather, each member of the literature search team was given their allotment of titles to filter through); and (2) if a study presented on several outcomes related to sweetness preference determinants (e.g., age and disease), the study was categorized based on the most relevant outcome to the primary objective of the study.

The “investigator checklist” for studies assessing sweetness preference determinants was developed to assess the methodological robustness of the studies; another appropriate tool could not be identified. Among the included studies, great variability in study design—and a lack of reporting of confounding factors and covariates—was evident. The objective of the “investigator checklist” tool was to highlight key areas for further consideration in the design of studies assessing sweetness preference. Future sweetness preference studies may benefit from leveraging this “investigator checklist”.

## 5. Conclusions

In this scoping review, determinants of sweetness preference were grouped into 12 distinct categories. Importantly, evaluation of ‘sweetness preference’ is predicated on the notion that ‘sweetness’ is detected by the individual first, with each individual potentially having a different threshold below which ‘sweetness’ would not even register. Overall, the studies identified in this scoping review were significantly heterogeneous with respect to the levels and ranges of sweetness provided and the methodology utilized to assess sweetness preference—all of which could have contributed to inconsistencies in the reported findings across studies. The lack of standardization in the field of ‘sweetness preference’ testing renders interpretation and generalizability across studies quite challenging and is certainly an area that requires further standardization. An “investigator checklist” was developed as a tool to assist in the identification and evaluation of study parameters that may be critical in sweetness preference assessments. This tool was developed as a result of the inconsistencies among the relevant studies in study design and other key areas that lacked standardization.

## Figures and Tables

**Table 1:** Outline of parameters included in the “investigator checklist”.

1	**Standardization of Procedures for Examiners**	
	1a	Smoking status
	1b	Avoidance of scented personal products (i.e., perfume/cologne use)
	1c	Avoidance of confectionary consumption (e.g., gums/mints) with strong flavors/scents
	1d	Avoidance of foods with strong odors
	1e	Standardization of questioning and information provided to subjects
2	**Standardization of Subject Characteristics**	
	2a	Age
	2b	Gender
	2c	BMI/body weight
	2d	Ethnicity/culture
	2e	For females, stage of the menstrual cycle, and/or pregnancy status
	2f	Health status (e.g., chronic disease state)
	2g	Health status (e.g., acute disease/health condition that could affect sweetness preference)
	2h	Medication use
*3*	**Standardization of Procedures for Subjects**	
	*3a*	Fasting; satiation status
	*3b*	Standardization of last meal consumed (NA: If subjects fasted overnight)
	*3c*	Smoking (i.e., including vaping, snuff, chewing tobacco, e-cigarettes, etc.)
	*3d*	Alcohol consumption/use of recreational drugs (e.g., cannabis)
	*3e*	Avoidance of scented personal products (i.e., perfume/cologne use)
	*3f*	Avoidance of confectionary (e.g., gums/mints) with strong flavors/scents; teeth brushing/use of mouthwash
	*3g*	Physical activity
	*3h*	Habitual use of non-caloric sweeteners (not applicable for caloric sweetener studies)
4	**Standardization of Sweetness Preference Testing**	
	4a	Clear reporting of sweetener or sweetened product tested
	4b	Clear reporting of sweetness concentrations tested
	4c	Vehicle composition across challenges changes only with regards to sweetness
	4d	Random order of challenges
	4e	Consistent volume/amount of each challenge
	4f	Consistent temperature of each challenge
	4g	Consistent presentation of each challenge (e.g., in opaque cups)
	4h	Consistent nature of each challenge (e.g., whole mouth rinse, complete ingestion, or swabbing of a particular area on the tongue)
	4i	Consistent duration of each challenge
	4j	Consistent time of day at which challenges were presented
	4k	Rinsing of palate between challenges
	4l	Consistent time interval between challenges
	4m	Control of environmental stimuli during sweetness testing

**Table 2:** Summary of findings: Effects of identified determinants on sweetness preference.

Sweetness Preference Determinant	Studies		Overall Findings
	No.	Consistent Findings?	
1. Age	9	Yes	• 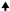 in childhood and elderly populations.
2. Dietary/Nutritional Factors	14	Some	• 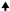 in fasted vs. satiated state.
			• Findings for sweetness preference based on dietary macronutrient composition or meal composition were inconsistent.
3. Reproductive Hormonal Factors	7	Some	• Possible inverse relationship: 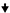 with higher levels of progesterone.
4. Genetics/Heritability	9	Some	• Heritability accounted for some of the variability in sweetness preference.
			• Inconsistent findings with respect to sweetness preference and PROP sensitivity.
5. Body Weight	11	Yes	• No association between sweetness preference and BMI status across age groups and genders.
6. Weight Loss	5	No	• Inconsistent findings with respect to the effects of weight loss on sweetness preference.
7. Sound	2	N/A	• Limited evidence to draw conclusions.
8. Personality Traits	5	No	• Inconsistent findings. Limited evidence to draw conclusions.
9. Ethnicity and Lifestyle			
• Different Ethnic Groups	5	Yes	• 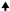 among westernized cultures compared to their Asian counterparts.
			• 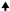 may be due to familiarization of foods.
• Traditional/Modern Lifestyles	3	Yes	• 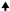 with a modern vs. traditional lifestyle.
			• 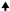 may be due to familiarization of foods.
• Physical Activity Levels	2	Yes	• 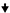 in individuals who are more vs. less physically active.
10. Previous Sweetness Exposure	6	No	• Inconsistent findings.
11. Disease			
• Neurological/Psychological	7	No	• Inconsistent findings among studies in subjects with a neurological or psychological disease.
• T2DM/GDM	3	No	• Limited evidence among studies in individuals with T2DM or GDM to draw conclusions.
Other	2	No	• Limited evidence among studies in individuals with other diseases (namely, PWS or end-stage renal disease) to draw conclusions.
12. Other Factors	8	N/A	• Study objectives and results were too diverse to draw conclusions.

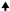
 = increased sweetness preference; 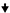 = decreased sweetness preference; BMI = body mass index; b/w = between; GDM = gestational diabetes mellitus; N/A = not applicable; No. = number; PROP = 6-n-propylthiouracil; PWS = Prader-Willi Syndrome; T2DM = type 2 diabetes mellitus; GDM = Gestational Diabetes Mellitus; vs. = versus.

## Supplementary Materials

The following are available online at https://www.mdpi.com/2072-6643/12/3/718/s1: Supplementary File S1: Table S1-1: Keywords used in the literature searches; Supplementary File S2: Table S2-1: Considerations in the design of sweetness preference studies: an “investigator checklist”; Supplementary File S3: Tables S3-1 to S3-12: Summary Tables; Supplementary File S4: Table S4-1: Application of the “investigator checklist”.

## Abbreviations

BMI: body mass index; GDM, gestational diabetes mellitus; GI, gastrointestinal; NA, not applicable; NNS, non-nutritive sweeteners; NS, nutritive sweetener; PD, Parkinson’s disease; PROP, 6-n-propylthiouracil; PWS, Prader-Willi Syndrome; T1R2, taste 1 receptor member 2; T1R3, taste 1 receptor member 3; T2DM, type 2 diabetes mellitus; T2R, bitter taste receptor; U.S., United States.
