# Supplementary material for: Determinants of Sweetness Preference: A Scoping Review of Human Studies
Source: Nutrients. 2020 Mar 8;12(3):718. doi: 10.3390/nu12030718 (PMC7146214; doi:10.3390/nu12030718)
Supplement: Supplementary file 1 [file nutrients-12-00718-s001.zip › Supplementary File S1 - Venditti et al., 2020.pdf]

Supplementary File S1

Table S1-1. Keywords used in the literature searches.

Keyword Type	Keywords used in the First Search[a,b,c]	Keywords used in the Second Search[b]
Sweeteners	ti,ab(sweetener or aspartame or Splenda or Equal or advantame or cyclamate or saccharin or sucralose or stevia or stevio* or rebaudioside or acesulfame or alitame or psicose or tagatose or allose or allulose or glycerol or erythritol or threitol or arabitol or xylitol or ribitol or mannitol or sorbitol or galactitol or fucitol or iditol or inositol or volemitol or isomalt or maltitol or lactitol or maltotriitol or maltotetraitol or polyglycitol or sugar or glucose or fructose or HFCS or galactose or disaccharide or syrup or honey or agave or sucrose or lactose or maltose or "flavor modifying properties" or "flavour modifying properties" or FMP or (caloric or sugar-sweetened or “sugar sweetened” or "non-caloric" or noncaloric or (sweet* near/3 (concentration or level))	Not applicable
Sweetness preference/ conditioning	ti,ab((sweet*) and (prefer* or liking or like or seek* or condition* or perceiv* or perception or craving or addiction or dependence or detection or recognition or wanting or consuming or receptor or taste or desire or motivation or consuming or flavor or flavour or intensity or sensitivity or adaptation))	ti,ab(effect* pre/15 (sweetness or “sweet taste”)
Human population	ti,ab(man or men or woman or women or human or subject or participant or volunteer or adult or people or person or individual or elder* or senior or geriatric or older or pregnan* or lactat* or breasfe* or breast-fe* or "breast fe*" or pediatric or paediatric or teen* or adolescen* or child* or boy or girl or toddler or baby or infant or neonate or student or panel*)	Not applicable
Irrelevant document types[d]	“Conference Abstract" or "Meeting Abstract" or "Conference paper" or "Patent" or "English Abstract" or "Thesis" or "Conference Paper" or "Conference Review" or "Editorial" or "Letter" or "Bulletin" or "Case Reports" or "Comment" or "Conference proceedings" or "Erratum" or "Book" or "Book chapter" or "Chapter" or "Meeting Poster" or "Twin Study" or "Video-Audio Media"	

[a] The literature searches were first conducted on 28 November 2017, and were updated on 1 October 2018).
[b] The asterisk is intended to allow flexibility in the word ending (*e.g.*, “adolescen*” would result in the identification of “adolescence” or “adolescent”). The function “near/X”, where X is a number, searches for the keywords preceding and following the near/X function that occur together within X number of words (*e.g.*, “sweet* near/3 concentration” would result in the identification of “sweetener concentration” or “concentration of sweeteners”). Using the “pre/X” function, the order of appearance of the keywords is defined such that the keyword preceding the pre/X function has to occur before the keyword following the function (*e.g.*, effect* pre/15 sweetness would result in the identification

Table S1-1. Keywords used in the literature searches.

Keyword Type	Keywords used in the First Search[a,b,c]	Keywords used in the Second Search[b]

of "effects of sweetness…" but not "…sweetness and its effects on…"). In this example, up to 15 terms can separate "effect*" and "sweetness" or "sweet taste".

[c] For an article to be identified, at least one term from each category of search terms had to appear in the title or abstract of the article.

[d] Articles categorized as irrelevant document types were not retrieved.
