# Supplementary material for: Determinants of Sweetness Preference: A Scoping Review of Human Studies
Source: Nutrients. 2020 Mar 8;12(3):718. doi: 10.3390/nu12030718 (PMC7146214; doi:10.3390/nu12030718)
Supplement: Supplementary file 1 [file nutrients-12-00718-s001.zip › Supplementary File S2 - Venditti et al., 2020.pdf]

Supplementary File S2: Considerations in the Design of Sweetness Preference Studies: An Investigator Checklist

Table S2-1. Considerations in the design of sweetness preference studies: an "investigator checklist".

	Parameter	Justification for addition to checklist	Additional considerations applied to the scoring parameter	Score		
				1 (Y)	0 (N, NR)	NA
**1**	**Standardization of Procedures for Examiners**[a]					
**1a**	Smoking status	The examiner's smoking status, and prior smoking, in the acute time period before testing, may affect the subject's perception of sweetness.				
**1b**	Avoidance of scented personal products (i.e., perfume/cologne use)	The examiner's use of scented personal products, may affect the subject's perception of sweetness.				
**1c**	Avoidance of confectionary consumption (e.g., gums/mints) with strong flavors/scents	The examiner's consumption of strong-smelling confectionary or gums may affect the subject's perception of sweetness.				
**1d**	Avoidance of foods with strong odors	The examiner's consumption of strong-smelling foods may affect the subject's perception of sweetness.				
**1e**	Standardization of questioning and information provided to subjects	If the examiner provides information to the participants regarding the assessment of sweetness of preference of investigational products, or regarding the procedure participants must follow, the language and method of provision should be standardized.				

Table S2-1. Considerations in the design of sweetness preference studies: an "investigator checklist".

	Parameter	Justification for addition to checklist	Additional considerations applied to the scoring parameter	Score 1 (Y)	Score 0 (N, NR)	Score NA
**2**	**Standardization of Subject Characteristics[b]**					
**2a**	Age	Age is a confounder in sweetness preference; young children and elderly individuals prefer more sweet foods.				
**2b**	Gender	To ensure balanced assessments, studies should report the gender of the included subjects.				
**2c**	BMI/body weight	Provision of information regarding the participants' weight/BMI status should be reported.	*General indication of body weight status* (e.g., *"healthy" or normal weight) was considered sufficient*			
**2d**	Ethnicity/culture	A person's ethnicity often determines his/her experience with foods and this experience may affect one's preference for foods and/or sweetness preference.	*NA if the determinant of sweetness preference that is being assessed in ethnicity or culture or if involves subjects from the same family* (e.g., *mother and infant pair)*			
**2e**	For females, stage of menstrual cycle, and/or pregnancy status.	Studies suggest that hormonal changes may affect one's sweetness preference.	*NA if the study included only males or children and/or adolescents prior to puberty.*			
**2f**	Health status (e.g., chronic disease state)	Identifying individuals which may have been diagnosed with a chronic disease (e.g., diabetes).	*NA if the health condition was the determinant of sweetness preference*			

Table S2-1. Considerations in the design of sweetness preference studies: an "investigator checklist".

	Parameter	Justification for addition to checklist	Additional considerations applied to the scoring parameter	Score 1 (Y)	0 (N, NR)	NA
**2g**	Health status (e.g., acute disease/health condition that could affect sweetness preference)	Identifying acute health conditions (e.g., a common cold) which may affect sweetness preference.				
**2h**	Medication use	Concomitant use of medication should be accounted for, as certain medications may lead to an ill after-taste and/or may affect one's overall health (e.g., nausea) and thereby affecting one's sweetness preference.				
**3**	**Standardization of Procedures for Subjects**[b]					
**3a**	Fasting; satiation status	A fasted or fed state will affect one's preference for sweet tasting foods (e.g., in a fasted state, preference for sweetness will increase, perhaps in an attempt to increase blood glucose levels rapidly); therefore, this parameter should be standardized.	*Acute standardized fasting should be quantified* (e.g., *all participants were fasted for 4 hours prior to testing).*<br><br>*Extended durations of fasting may instead be qualified* (e.g., *an "overnight fast" may be acceptable, rather than indicating 8 or 12 hours in duration).*			
**3b**	Standardization of last meal consumed (NA: if subjects are fasted overnight)	Nutrient content of the previous meal may affect desire to eat and also sweetness preference.	*Longer fasting periods reduce the likelihood of a previous meal affecting a sweetness preference and would therefore deem this parameter NA.*			

Table S2-1. Considerations in the design of sweetness preference studies: an "investigator checklist".

	Parameter	Justification for addition to checklist	Additional considerations applied to the scoring parameter	Score 1 (Y)	Score 0 (N, NR)	Score NA
**3c**	Smoking (i.e., including vaping, snuff, chewing tobacco, e-cigarettes etc.)	Standardization of the use of tobacco products (i.e., ensuring consistent use among participants and/or including parameters to exclude subjects using these products); the use of tobacco products may affect one's tasting preferences and therefore sweetness preference.				
**3d**	Alcohol consumption/use of recreational drugs (e.g., cannabis)	Standardization of alcohol consumption and/or use of recreational drugs.				
**3e**	Avoidance of scented personal products (i.e., perfume/cologne use)	The use of scented personal products, may affect a subject's perception of sweetness.				
**3f**	Avoidance of confectionary (e.g., gums/mints) with strong flavors/scents; teeth brushing/use of mouthwash	The consumption of strong-smelling confectionary or gums may affect a subject's perception of sweetness.				
**3g**	Physical activity	Standardization of physical activity prior to testing, as the effect of exercise on a subjects' taste preference is not known.	*An example of standardization of this parameter may be: subjects should refrain from extensive physical activity within 4 hours of testing. Recording subject habitual physical activity does not satisfy this parameter.*			

Table S2-1. Considerations in the design of sweetness preference studies: an "investigator checklist".

	Parameter	Justification for addition to checklist	Additional considerations applied to the scoring parameter	Score		
				1 (Y)	0 (N, NR)	NA
**3h**	Habitual use of non-caloric sweeteners (not applicable for caloric sweetener studies)	Personal preference related to the taste of non-caloric sweeteners may affect the evaluation of one's sweetness preference.	*Some persons dislike certain non- caloric sweeteners; therefore, the inclusion of such persons in an experiment of sweetness preferenceis not appropriate.*			
**4**	**Standardization of Sweetness Preference Testing**[a]					
**4a**	Clear reporting of sweetener or sweetened product tested	In order to assess the effect of an investigational product, the sweetener used (i.e., name of caloric or non-caloric sweetener) must be adequately described.	*It is not sufficient to indicate the name of a beverage brand (e.g., Diet Pepsi); the type of sweetener used in the product must be clearly defined.*			
**4b**	Clear reporting of sweetness concentrations tested	In order to assess the effect of an investigational product, the concentration of sweetener used must be adequately described.	*For example, the provision of 250 ml of water sweetened with 0.5%, 1.0% and 1.5% sucralose.*			
**4c**	Vehicle composition across challenges changes only with regards to sweetness	To ensure independent effects of the sweetener are assessed, for each challenge, the composition of the vehicle must only change with respect to the concentration of or the sweetener used.	*Addition of any other ingredients to an investigational product, may deem it difficult to determine if the sweetness preferences identified are simply due to the sweetener or the concentration of sweetness, rather than the additional ingredient itself.*			
**4d**	Random order of challenges	Challenges should be provided in random order to account for a sequence effect.				

Table S2-1. Considerations in the design of sweetness preference studies: an "investigator checklist".

	Parameter	Justification for addition to checklist	Additional considerations applied to the scoring parameter	Score		
				1 (Y)	0 (N, NR)	NA
**4e**	Consistent volume/amount of each challenge	Standardization of the amount/volume of the challenge provided to subjects. Provision of investigational products *ad libitum* would not score a point for this parameter.	*Regardless of the amount of the challenge consumed, if researchers provided consistent volumes of the investigational products to participants, this parameter would be satisfied.*			
**4f**	Consistent temperature of each challenge	All investigational products should be provided to participants ensuring consistent temperature.				
**4g**	Consistent presentation of each challenge (e.g., in opaque cups)	To ensure consistency in the provision of products, and to ensure blinding of the investigational products, presentation of each challenge should be consistent.				
**4h**	Consistent nature of each challenge (e.g., whole mouth rinse, complete ingestion, or swabbing of particular area on tongue)	To ensure comparisons are adequately assessed within a study, all challenges must be provided to each subject in a consistent manner.				
**4i**	Consistent duration of each challenge	To reduce inter and intra-subject variability, participants should be provided with challenges for consistent durations.				
**4j**	Consistent time of day at which challenges were presented	Provision of challenges at inconsistent times of day may affect the participants' sweetness preference.	Consistent time periods should be provided for provision of challenges (e.g., 9-11am). Broad descriptions should be avoided (e.g., morning).			

Table S2-1. Considerations in the design of sweetness preference studies: an "investigator checklist".

	Parameter	Justification for addition to checklist	Additional considerations applied to the scoring parameter	Score 1 (Y)	Score 0 (N, NR)	Score NA
**4k**	Rinsing of palate between challenges	This parameter is most important when challenges numerous are provided in a sequential fashion, over a short duration. To ensure no residual taste or effect is "carried over" to the next challenge, appropriate palate rinsing should be indicated.				
**4l**	Consistent time interval between challenges	To ensure each participant experiences the same time interval between challenges – this ensures time to remember challenge tastes is consistent for rating preferences.				
**4m**	Control of environmental stimuli during sweetness testing	The location of the testing should be consistent for each participant, limiting external factors that may affect the rating of sweetness preference (e.g., external smells, extreme lighting, distracting sounds).				

N = no; NA = not applicable; NR = not reported; Y = yes.

[a] The purpose of standardization is to reduce the effects of potential confounders across all subjects.

[b] The purpose of standardization is to reduce the effects of potential confounders between the study groups (e.g., the group of subjects with the potential determinant *versus* the group of subjects without the potential determinant). Therefore, the parameters in the Standardization of Subject Characteristics should not only be reported, but also be similar between groups.
