# Supplementary material for: Determinants of Sweetness Preference: A Scoping Review of Human Studies
Source: Nutrients. 2020 Mar 8;12(3):718. doi: 10.3390/nu12030718 (PMC7146214; doi:10.3390/nu12030718)
Supplement: Supplementary file 1 [file nutrients-12-00718-s001.zip › Supplementary File S3 - Venditti et al., 2020.pdf]

Supplementary File S3

List of Summary Tables

Table S3-1. Studies in which age was assessed as a sweetness preference determinant (n=9)....2
Table S3-2. Studies in which dietary/nutritional factors were assessed as sweetness preference determinants (n=14)....7
Table S3-3. Studies in which reproductive hormonal factors were assessed as sweetness preference determinants (n=7)....15
Table S3-4. Studies in which genetic/heritable factors were assessed as sweetness preference determinants (n=9). 18
Table S3-5. Studies in which body weight status was assessed as a sweetness preference determinant (n=11). 22
Table S3-6. Studies in which weight loss was assessed as a sweetness preference determinant (n=5)....26
Table S3-7. Studies in which sound was assessed as a sweetness preference determinant (n=2)....28
Table S3-8. Studies in which personality traits were assessed as sweetness preference determinants (n=5). 29
Table S3-9. Studies in which ethnicity and lifestyle were assessed as sweetness preference determinants (n=10). 31
Table S3-10. Studies in which previous exposure to sweets was assessed as a sweetness preference determinant (n=6). 37
Table S3-11. Studies in which disease was assessed as a sweetness preference determinant (n=12)....40
Table S3-12. Studies in which other factors were assessed as sweetness preference determinants (n=8). 45

Table S3-1. Studies in which age was assessed as a sweetness preference determinant (n=9).

Reference	Study Population (Sample Size)			Food Delivery Matrix	Sweetener	Sweetness		Method of Assessment	Results
	Children/ Adolescents	Adults	Elderly			Levels	Number of Levels		
**Studies Comparing the Sweetness Preferences of Children or Adolescents and Young Adults (n=4)**									
De Graaf and Zandstra (1999) [35]	Children (8-10 y; n=30 M+F)<br><br>Adolescents (14-16 y; n=30 M+F)	Young adults (20-25 y; n=30 M+F)	NA	Water	Sucrose	1%, 4.8%, 6.9%, 14.4%, 20.8%, 30.0% (wt/vol)	6	5-point category scale; rank order of preference	↑ (SS): children *v.* adolescents and children *v.* young adults; ↑ (SS) adolescents *v.* young adults
				Orangeade	Sucrose	1%, 4.8%, 6.9%, 14.4%, 20.8%, 30.0% (wt/vol)	6	5-point category scale; rank order of preference	↑ (SS): children *v.* young adults
Liem and De Graaf (2004) [36]	Children (n=59 M+F)	Young adults (n=46 M+F)	NA	Orangeade	Citric acid (sweetness suppressant)<br><br>Sucrose	*14.4%[a] (wt/vol)*<br><br>0.42 M	1	Preference ranking (1=most preferred; 7=least preferred)	↑ (SS): children *v.* young adults
				Yoghurt	Citric acid (sweetness suppressant)<br><br>Sucrose	*14.4%[b] (wt/vol)*<br><br>0.42 M	1	Ranked preference (1=most preferred; 7=least preferred)	NR
Desor et al. (1975) [33]	Adolescents (9-15 y; n=618 M+F)	Adults (18-64 y; 140 M+F)	NA	Solution	Sucrose	*2.6%, 5.1%, 10.3%, 20.5% (wt/vol)*<br><br>[0.075M, 0.15M, 0.30M, 0.60M]	4	Ranked preference (from most to least preferred)	↑ for 0.60M (SS): adolescents *v.* adults

Table S3-1. Studies in which age was assessed as a sweetness preference determinant (n=9).

Reference	Study Population (Sample Size)			Food Delivery Matrix	Sweetener	Sweetness		Method of Assessment	Results
	Children/ Adolescents	Adults	Elderly			Levels	Number of Levels		
Desor and Beauchamp (1987) [34]	Adolescents (11-15 y; n=44 M+F)	Young adults (19-25 y; n=44) – follow up using original participants	NA	Water	Sucrose	*2.6%, 5.1%, 10.3%, 20.5% (wt/vol)*<br><br>[0.075, 0.150, 0.300, and 0.600 M]	4	Ranked preference (from most to least preferred)	↑ (SS): adolescents *v.* young adults
**Studies Comparing the Sweetness Preferences of Young Adults and Elderly (n=1)**									
Mojet et al. (2005) [37]	NA	Young adults (19-33 y; n=21 M+F)	Elderly (60-75 y; n=21 M+F)	Iced tea	Sucrose	5.395%-34.038% wt/vol [53.95-340.38 g/L]	5	Liking rating (9-point scale: very little = to the left; very much = to the right)	↑ (SS): elderly *v.* young
				Iced tea	Aspartame	0.015%-0.092% wt/vol [0.15-0.92 g/L]	5	Liking rating (9-point scale: very little = to the left; very much = to the right)	Age by gender effect (SS): ↑ in elderly M *v.* elderly F and young M, but not young F

Table S3-1. Studies in which age was assessed as a sweetness preference determinant (n=9).

Reference	Study Population (Sample Size)			Food Delivery Matrix	Sweetener	Sweetness		Method of Assessment	Results
	Children/ Adolescents	Adults	Elderly			Levels	Number of Levels		
**Studies Comparing the Sweetness Preference of Children and their Mothers (n=4)**									
Bobowski and Mennella (2017) [41]	Children (7-14 y; n=34 GD NR)	Mothers (n=26F)	NA	Water	Sucrose	*10.3%, 20.5%, 35.9% (wt/vol)* [300, 600, 1,050 mM]	3	Liking rating (3-point facial hedonic scale: super bad; neutral; super good)	NSD
				Water	Sucralose	*0.02%, 0.08% (wt/vol)* [0.40, 2.0 mM]	2	Liking rating (3-point facial hedonic scale: super bad; neutral; super good)	NSD
				Water	Aspartame	*0.01%, 0.06% (wt/vol)* [3.0, 10.0 mM]	2	Liking rating (3-point facial hedonic scale: super bad; neutral; super good)	NSD
				Water	Sucrose	*10.3%, 20.5%, 35.9% (wt/vol)* [300, 600, 1,050 mM]	3	Liking rating (5-point facial hedonic scale: super bad; neutral; super good)	↑ for 1,050 mM (SS): children *v.* mothers
				Water	Sucralose	*0.02%, 0.08% (wt/vol)* [0.40, 2.0 mM]	2	Liking rating (5-point facial hedonic scale: super bad; neutral; super good)	↑ for 2 mM (SS): children *v.* mothers
				Water	Aspartame	*0.01%, 0.06% (wt/vol)* [3.0, 10.0 mM]	2	Liking rating (5-point facial hedonic scale: super bad; neutral; super good)	↑ for 10 mM (SS): children *v.* mothers

Table S3-1. Studies in which age was assessed as a sweetness preference determinant (n=9).

Reference	Study Population (Sample Size)			Food Delivery Matrix	Sweetener	Sweetness		Method of Assessment	Results
	Children/ Adolescents	Adults	Elderly			Levels	Number of Levels		
				Water	Sucrose	*3.1%, 6.2%, 12%, 24%, 36% (wt/vol)*<br><br>[90, 180, 350, 700, 1,050 mM]	5	Forced-choice tracking: (asked which of the two sucrose solutions they preferred)	🡅 (SS): children *v.* mothers
				Water	Sucralose	*0.005%, 0.01%, 0.02%, 0.05%, 0.06% (wt/vol)*<br><br>[0.13, 0.26, 0.5, 1.17, 1.50 mM]	5	Forced-choice tracking: (asked which of the two sucralose solutions they preferred)	🡅 (SS): children *v.* mothers
Mennella et al. (2012) [39]	Children (5-10.9 y; n=84 M+F)	Mothers (21-52 y; n=67 F)	NA	Water	Sucrose	3%, 6%, 12%, 24%, 36% (wt/vol)	5	Forced-choice tracking: (asked which of the two sucrose solutions they preferred)	🡅 (SS): children *v.* mothers
				Vanilla pudding	Sucrose	13.4%, 24.1%, 36.2% (wt/wt)	3	Forced-choice tracking: (asked which of the two sucrose solutions they preferred)	🡅 (SS): children *v.* mothers (p=0.05, stated to be SS in publication)
Mennella et al. (2014) [40]	Children (5-10 y; n=101 GD NR)	Mothers (n=76 F)	NA	Water	Sucrose	3-36% (wt/vol)	5	Monell two-series, forced-choice, paired-comparison tracking: (asked which of the two sucrose solutions they preferred)	🡅 (SS): children *v.* mothers

Table S3-1. Studies in which age was assessed as a sweetness preference determinant (n=9).

Reference	Study Population (Sample Size)			Food Delivery Matrix	Sweetener	Sweetness		Method of Assessment	Results
	Children/ Adolescents	Adults	Elderly			Levels	Number of Levels		
Pepino and Mennella (2005) [38]	Children (5-10 y; n=164 GD NR)	Mothers (n=193 F)	NA	Water	Sucrose	3%, 6%, 12%, 24%, 36% (wt/vol)	5	Forced-choice tracking: (asked which of the two sucrose solutions they preferred)	↑ (SS): children *v.* mothers

↑ = increased sweetness preference; F = females; GD = gender distribution; M = males; n = number; NA = not applicable; NR = not reported; NSD = no significant differences; SS = statistically significant; *v.* = versus; vol = volume; wt = weight; y – years;

[a] All solutions contained 0.42M sucrose and contained varying levels of citric acid (0.0M, 0.009M, 0.013M, 0.020M, 0.029M, 0.043M, 0.065M) as citric acid levels increase, perceived sweetness decreases, as citric acid is a sweetness suppressant.

[b] All yogurts contained 0.42 M sucrose and contained varying levels of citric acid (0.0M, 0.027M, 0.038M, 0.056M, 0.081M, 0.12M, 0.17M).

Table S3-2. Studies in which dietary/nutritional factors were assessed as sweetness preference determinants (n=14).

Reference<br><br>Study Design	Study Population (Sample Size)[a]<br>Control	 Comparator(s)	Food Delivery Matrix	Sweetener	Sweetness<br>Levels	 Number of Levels	Method of Assessment	Results
**Studies in which Sweetness Preference During Fed versus Fasted States were Assessed (n=6 studies)**								
Fantino et al. (1983) [44]<br><br>Before-and-after	Before the Hajba[b], in a fasted state and after a glucose load (n=8 F)	After the Hajba[b], in a fasted state and after a glucose load	Water	Sucrose	*2.1%, 4.3%, 8.6%, 17.1%, 34.2%* (wt/vol)<br><br>[0.0625, 0.125, 0.25, 0.5, 1.0 M]	5	Pleasantness rating (-2=very unpleasant; +2=very pleasant); assessed – before and after the Hajba – in a fasted state and every 15 min for 75 min after the ingestion of 200 mL of 1.4 M glucose solution (50 g of glucose in 200 mL of water)	SS ↓ post-Hajba *v.* pre-Hajba (for fasted state only)<br><br>SS ↓ after glucose load *v.* fasting, both pre- and post-Hajba
Laeng et al. (1993) [46]<br><br>P	Sated adults (who ate within 2 h; n=NR, M+F)[c]	Hungry adults (who last ate ≥2 h ago)[c]	Lime Kool Aid	Sucrose	4.5%, 9%, 18%, 36% (wt/vol)	4	Pleasantness rating (-4=extremely unpleasant; +4=extremely pleasant)	SS ↓ in satiated *v.* hungry; further analyses revealed a gender effect, with SS ↓ noted for sated F *v.* hungry F, but not sated M *v.* hungry M

Table S3-2. Studies in which dietary/nutritional factors were assessed as sweetness preference determinants (n=14).

Reference<br><br>Study Design	Study Population (Sample Size)[a]		Food Delivery Matrix	Sweetener	Sweetness		Method of Assessment	Results
	Control	Comparator(s)			Levels	Number of Levels		
Looy and Weingarten (1991) [45]<br><br>X	Satiated adults (who consumed a meal 30 min before testing; n=28 M+F)	Hungry adults (who were fasted for 18 h)	Water	Sucrose	*1, 1.7, 3.4, 5.5, 7.2, 10.6, 14.4, 21.2, 28.4% (wt/vol)*<br><br>[0.03, 0.05, 0.10, 0.16, 0.21, 0.31, 0.42, 0.62, 0.83 M]	9	200mm Hedonic Scale (-100mm=disliked very much; +100mm=liked very much)	SS group X metabolic state interaction; SS ↑ in sweet dislikers when hungry *v.* satiated, but NSD in sweet likers when hungry *v.* satiated
Moskowitz et al. (1976) [43]<br><br>P	Hungry adults (fasted overnight for 14 h; n=16 M)	Satiated adults (who consumed breakfast; n=8 M)<br><br>Satiated adults (who consumed lunch; n=8 M)<br><br>Satiated adults (after an oral glucose load; n=8 M)	Water	Glucose	*1, 2.1, 4.3, 8.6, 17.1, 34.2, 68.5% (wt/vol)*<br><br>[0.03, 0.06, 0.125, 0.25, 0.5, 1.0, 2.0 M]	7	Pleasantness rating (1=extremely disliked; 6=extremely liked)	A breakpoint at 1.0 M glucose (beyond which there was a ↓ in the likeness of the solutions) was observed in all groups, except those tested after a glucose load, who showed ↑ for increasing glucose solutions beyond 1 M (SS NR).

Table S3-2. Studies in which dietary/nutritional factors were assessed as sweetness preference determinants (n=14).

Reference<br><br>Study Design	Study Population (Sample Size)[a]		Food Delivery Matrix	Sweetener	Sweetness		Method of Assessment	Results
	Control	Comparator(s)			Levels	Number of Levels		
Pangborn (1959) [42]<br><br>P (Consumer study)<br><br>X (Laboratory study)	Consumer study (n=11,456 M+F)		Cling peaches	Sucrose	24.24%, 26.95%, 26.98%, 27.59%, 31.20%, 31.51%, 36.77% (wt/wt)<br><br>(24.24, 26.95, 26.98, 27.59, 31.20, 31.51, 36.77 (Brix)	7	Series of paired comparisons (asked which of the two adjacent peaches they preferred)	NSD in sweetness preference when the participants were segregated according to their reported level of hunger
	Laboratory study fed state (n=8 M+F)	Fasted state (nothing consumed after breakfast until 4:30 pm)	Apricot nectar	Sucrose	8%, 9%, 10%, 11%, 12% (wt/vol)	5	Series of paired comparisons (asked which of the two adjacent apricot nectars they preferred)	NSD in sweetness preference when panelists were in a fed *v.* fasted state
Martin et al. (2016) [47]<br>X	Fasted adults (who fasted for 3 h prior to testing; n=40 M+F)	Sated adults (20 min after consuming lunch)	Gelatinous desserts	Truvia, Truvia + Sucralose	Truvia: 0%, 2%, 4%, 16%<br><br>Truvia + sucralose: 16% + 0.2%	5	100mm Preference Rating (0=extremely dislike; 100=extremely like)	SS main effect of hunger, such that there was a ↑ with increased hunger

Table S3-2. Studies in which dietary/nutritional factors were assessed as sweetness preference determinants (n=14).

Reference<br><br>Study Design	Study Population (Sample Size)[a]			Food Delivery Matrix	Sweetener	Sweetness		Method of Assessment	Results
	Control	Comparator(s)				Levels	Number of Levels		
**Studies in which the Effects of Different Meals/Diets/Dietary Habits on Sweetness Preference were Assessed (n=8)**									
Divert et al. (2017) [51]<br><br>Cross-sectional Observational	Children (7-12 y; n=126 M+F)<br><br>Relationship b/w sweetness preference and sweet drink consumption, candy and snack consumption, cereal, dairy, and fruit product consumption, added sugar exposure			Water	Sucrose	3.1, 6.2, 12, 24, 35.9%	5	9-point hedonic scale (1= do not like it at all; 9=like it a lot)	SS +ve associations b/w sweetness preference and candy and snack consumption
				Syrup	Sucrose	0, 1.3, 2.5, 8.7, 14.5%	5	9-point hedonic scale (1= do not like it at all; 9=like it a lot)	
				Milk	Sucrose	4.8, 7.2, 13.9, 22, 35.8%	5	9-point hedonic scale (1= do not like it at all; 9=like it a lot)	
Garneau et al. (2018) [50]<br><br>Cross-sectional Observational	Sweet likers (n=218 adults M+F)	Sweet neutrals (n=377 adults M+F)	Sweet dislikers (n=55 adults M+F)	Water	Sucrose	0, 2.4, 4.3, 7.7, 13.7% (wt/vol)	5	100-mm VAS (dislike extremely to like extremely)	Compared to sweet dislikers, sweet likers and neutrals has SS greater intakes of sweetened juice and sweetened tea; however, there were NSD b/w sweet likers and dislikers in total energy intake or water.
	Relationship b/w sweetness preference (assessed using the sucrose-water solutions) and habitual beverage intake was assessed.								

Table S3-2. Studies in which dietary/nutritional factors were assessed as sweetness preference determinants (n=14).

Reference<br><br>Study Design	Study Population (Sample Size)[a]		Food Delivery Matrix	Sweetener	Sweetness		Method of Assessment	Results
	Control	Comparator(s)			Levels	Number of Levels		
Ashi et al. (2017) [52]<br>Cross-sectional observational	Children (13-15 y; n=225 M+F)<br><br>Relationship b/w number of main meals, snacks, and sweet intake occasions *v.* sweetness preference. Also, relationship b/w number of sweet foods consumed and sweetness preference.		Water	Sucrose	*0.2% to 82.1% (wt/vol)*<br><br>[1.62 to 821.52 g/L]	10	Selection of the most preferred solution	SS -ve correlation b/w sweet taste preference and the number of main meals consumed (r=-0.480; p≤0.001)<br><br>SS +ve correlation b/w sweet taste preference and the number of snack occasions (r=0.286; P≤0.001)<br><br>SS +ve correlation b/w sweet taste preference and the number of sweet intake occasions (r=0.288; P≤0.001)

Table S3-2. Studies in which dietary/nutritional factors were assessed as sweetness preference determinants (n=14).

Reference<br><br>Study Design	Study Population (Sample Size)[a]			Food Delivery Matrix	Sweetener	Sweetness		Method of Assessment	Results
	Control	Comparator(s)				Levels	Number of Levels		
Pangborn and Giovanni (1984) [49]<br><br>P	Adults with low sweet intake (n=14 M+F)	Adults with medium sweet intake (n=26 M+F)	Adults with high sweet intake (n=11 M+F)	Lemonade	Sucrose	4%, 6%, 8%, 10%, 14%, 20%, 30% (wt/vol)	7	10cm Hedonic Rating (0cm=dislike extremely; 10cm=like extremely)	SS ↑ in subjects with a high *v.* medium *v.* low sweet intake (all groups were SS different from each other)
Tatano et al. (2016) [32]<br><br>Case-control	NF; habitual fat intake <25% of TDEI (n=116 M+F adults)[d]	HF; habitual fat intake ≥25% of TDEI (n=116 M+F adults)[d]		NA	NA	NA	NA	100mm VAS (sweet food cravings)	SS ↑ in habitual consumers of HF *v.* NF diets
Vazquez et al. (1982) [53]<br><br>P	Well-nourished infants (n=53 M+F)	Malnourished infants (tested ≤5 d after admission; n=20 M+F)<br><br>Malnourished infants (tested ≥6 d after admission; n=45 M+F)		Water	Sucrose	*0%, 6.9%, 13.7% (wt/vol)*<br><br>[0, 0.2, 0.4 M]	3	Summative volume of solution ingested (mL)	NSD

Table S3-2. Studies in which dietary/nutritional factors were assessed as sweetness preference determinants (n=14).

Reference<br><br>Study Design	Study Population (Sample Size)[a]		Food Delivery Matrix	Sweetener	Sweetness		Method of Assessment	Results
	Control	Comparator(s)			Levels	Number of Levels		
Wise et al. (2016) [48]<br><br>P	Habitual diet (n=13 M+F adults)	Low sugar diet (n=16 M+F adults)	Vanilla pudding	Sucrose	0%, 6.6%, 11%, 25%, 31%, 40%, 47%, 52% (wt/wt)	8	Pleasantness rating (-11=very unpleasant; +11=very pleasant)	NSD
			Raspberry beverage	Sucrose	0%, 2.5%, 5%, 7.5%, 10%, 12.5%, 16%, 19%, 25% (wt/wt)	9	Pleasantness rating (-11=very unpleasant; +11=very pleasant)	NSD
Zhou *et al*. (2015) [31]<br><br>R, X	Adults (n= 65 M+F)		NA	NA	NA	NA	$AUC_{0\text{-}120\ min}$ using a 100mm VAS (sweetness desire), which was assessed after consuming 1of 6 standardized lunches on 6 different test days, each separated by 1 wk[e]	SS ↑ in F after consuming the high-meat/low-rice and the low vegetable meals

Table S3-2. Studies in which dietary/nutritional factors were assessed as sweetness preference determinants (n=14).

Reference<br><br>Study Design	Study Population (Sample Size)[a]		Food Delivery Matrix	Sweetener	Sweetness		Method of Assessment	Results
	Control	Comparator(s)			Levels	Number of Levels		

🡅 = increased sweetness preference; 🡇 = decreased sweet taste preference; +ve = positive; -ve = negative; AUC= area-under-curve; BMI = body mass index; b/w = between; d = days; F = females; h = hours; HF = high fat; M = males; min = minutes; n = number of subjects; NA = not applicable; NF = normal fat; NR = not reported; NSD = no significant differences; P = parallel; R = randomized; SS = statistically significant; TDEI = total daily energy intake; *v.* = versus; VAS = visual analogue scale; vol = volume; wk = week; wt = weight; X = crossover; y = years.

[a] Sample sizes are reported for the comparator(s) only if the study was P in design.

[b] The Hajba is an over-feeding regimen, lasting from 12 to 16 weeks, that is imposed on young girls before marriage, to achieve an obesity, which is regarded as aesthetically pleasing.

[c] The numbers of "sated" and "hungry" participants were NR; however, the total n in the study was 57 (28M + 29F).

[d] Subjects in the 2 groups were matched for gender, age, and BMI. The study is described as having a randomized, controlled, crossover design during which subjects consumed, on 6 different occasions, 1 of 6 different meals, and for 5 hours thereafter, cravings for sweets using a VAS were assessed; however, the results reported were relative to habitual fat consumption (and were "regardless of the meals consumed", and so the comparison is more aligned with a case-control-design.

[e] The test lunches included: (i) a low energy density (control) meal; (ii) high-meat/low-rice meal; (iii) a medium-fat/low-vegetable meal; (iv) low-vegetable meal; (v) high-fat meal; or (vi) high-fat/low-vegetable meal.

Table S3-3. Studies in which reproductive hormonal factors were assessed as sweetness preference determinants (n=7).

Reference	Study Population (Sample Size)		Food Delivery Matrix	Sweetener	Sweetness		Method of Assessment	Results
Study Design					Levels	Number of Levels		
Dippel and Elias (1980) [55]<br><br>P	Non-pregnant, users of high-progestin OC (n=15F)	Non-pregnant, users of low-progestin OC (n=15 F)	Water	Sucrose	*5.1, 10.3, 20.5, 41.1% (wt/vol)*<br><br>[0.15, 0.30, 0.60, and 1.2 M]	4	Ranked the test solutions in order from most to least preferred	SS ↑ for 0.60 M and 1.2 M in low-progestin OC users *v.* high-progestin OC users<br><br>NSD in sweetness preference between the 3 trimesters of pregnancy<br><br>SS ↓ in pregnant *v.* non-pregnant women
	Non-pregnant, non-users of OCs (n=75 F)	Pregnant, (n=60 F)						
Tucci et al (2010) [27]<br><br>P	Non-users of OC (n=26 F)	Users of OC (n=29 F)	Jelly babies, milk chocolate, digestive cookies	NA	NA	NA	Amount of sweet foods consumed	NSD
Bowen and Grunberg (1990) [25]<br><br>P	Follicular phase (n=18 F)	Luteal phase (n=19 F)	Cake, chocolate, gum drops	NA	NA	NA	Amount of sweet foods consumed	SS ↑ during the luteal *v.* follicular phase

Table S3-3. Studies in which reproductive hormonal factors were assessed as sweetness preference determinants (n=7).

Reference<br><br>Study Design	Study Population (Sample Size)	Food Delivery Matrix	Sweetener	Sweetness		Method of Assessment	Results
				Levels	Number of Levels		
Frye et al. (1994) [59]<br><br>X (each subject was tested weekly for 4 weeks during the menstrual cycle)	Normal-weight women (n=25 F)	Dairy milk	Sucrose	0, 5, 10, 20%	4	160-mm scale (0 = dislike, 160 = like)	SS effect of phase X order, such that there was ↑ when testing began for women in luteal or menstrual weeks of the menstrual period.
Pliner and Fleming (1983) [57]<br><br>X (each subject was tested at the midpoint of the follicular and luteal phases)	Non-users of OC with normal menstrual cycle (n=34 F)	Water	Sucrose	2.5, 5, 10, 20, 40%	5	5-point scale (-2 = dislike, +2 = like)<br><br>The solutions were rated before and 15 or 30 min after the subjects consumed 50 g of glucose in 200 ml of aqueous solution.	SS ↓ after glucose load during luteal phase but not during follicular phase

Table S3-3. Studies in which reproductive hormonal factors were assessed as sweetness preference determinants (n=7).

Reference<br><br>Study Design	Study Population (Sample Size)	Food Delivery Matrix	Sweetener	Sweetness		Method of Assessment	Results
				Levels	Number of Levels		
Wright and Crow (1973) [56]<br><br>P	Non-users of OC (n=94 F)	Water	Sucrose	2.5, 5, 10, 20, 40%	5	5-point scale (-2 = dislike, +2 = like)<br><br>The solutions were rated before, 10 min after, and 1 h after drinking 200 ml of a 25% glucose solution.	SS ↓ during the luteal phase *v.* other phases<br><br>SS ↓ 10 min after the glucose load at all phases except ovulation<br><br>SS ↓ 1 h after the glucose load at all phases
Coldwell et al. (2009) [54]<br><br>P	Adolescents (n=143 M+F)	Water	Sucrose	*1.9 to 34.2%* (wt/vol) *(0.25 log-steps)*<br><br>[0.056 to 1 M]	6	5-point hedonic scale (1= dislike, 5 like)	NSD between puberty stages

↑ = increased sweetness preference; ↓ = decreased sweetness preference; F = females; h = hour; M = males; min = minutes; n = number; NA = not applicable; NSD = no significant differences; OC = oral contraceptive; P = parallel; SS = statistically significant; *v.* = versus; vol = volume; wt = weight; X = crossover

Table S3-4. Studies in which genetic/heritable factors were assessed as sweetness preference determinants (n=9).

Reference	Study Population (Sample Size)	Food Delivery Matrix	Sweetener	Sweetness		Method of Assessment	Results
				Levels	Number of Levels		
**Studies Conducted Amongst Twins or Family Members (n=3)**							
Bretz et al. (2006) [60]	Children (n=230 M+F; 44 pairs of monozygotic twins and 71 pairs of dizygotic twins)	Grape juice	Sucrose	*5.1%, 9.9%, 20.2%, 26.4%, 40.0%* (wt/vol)<br><br>[0.15, 0.29, 0.59, 0.77, 1.17 M]	5	Sweetness Preference Inventory; Face Scale ranking (0 – frown; 1- neutral; 2 – smile) and sucrose sweetness preference score (composite score based on Face Scale ranking; range of 0-10)	SS influence of heritability on sweetness preference
Keskitalo et al. (2007a) [61]	Adults (17 to 80 y) monozygotic twins (n=303 F; 149 complete pairs) and dizygotic twins (360 F; 175 complete pairs)	Water	Sucrose	20% (wt/vol)	1	Preference rating, 120-mm vertical labelled affective magnitude scale (0 – greatest imaginable dislike; 120 – the strongest imaginable sensation)	In monozygotes and dizygotes, 48% and 26%, respectively, of the variability in sweetness preference was explained by genetic factors (presumed to be SS; but, SS NR)

Table S3-4. Studies in which genetic/heritable factors were assessed as sweetness preference determinants (n=9).

Reference	Study Population (Sample Size)	Food Delivery Matrix	Sweetener	Sweetness Levels	Sweetness Number of Levels	Method of Assessment	Results
Keskitalo et al. (2007b) [62]	Adults (n=146 M+F) from 26 Finnish families	Water	Sucrose	3.0%, 7.5%, 18.75% (wt/vol)	3	Sweet pleasantness rating, 12.5-cm labelled affective magnitude scale (0.0 – extremely unpleasant; 12.5 – greatest imaginable pleasantness)	Preference for the 2 strongest sucrose solutions (7.5% and 18.75%) yielded the highest heritability estimates (29.2% and 41%, respectively; both SS)[a]
**Study Conducted in Individuals with Different Leptin and Leptin Receptor Gene Polymorphisms (n=1)**							
Mizuta *et al.*, 2008 [26]<br><br>Cross-sectional Observational	Citizens (n=3,663 M+F; GD NR)<br><br>Various genotypes of the leptin gene and leptin receptor gene	NA	NA	NA	NA	Questioned whether “Do you like things that taste sweet?” (5-point scale; 1=No, I hate them, 5=Yes, I love them)<br><br>Rating of 5 were considered to have a “sweet” tooth.	Leptin gene A19G and leptin receptor gene R109K polymorphisms were SS associated with having a sweet tooth.

Table S3-4. Studies in which genetic/heritable factors were assessed as sweetness preference determinants (n=9).

Reference	Study Population (Sample Size)			Food Delivery Matrix	Sweetener	Sweetness		Method of Assessment	Results
						Levels	Number of Levels		
**Studies Conducted According to PROP Responsiveness (n=5)**									
Drewnowski et al. (1998) [67]	Adult PROP non-tasters (n=39 F)	Adult PROP medium tasters (n=48 F)	Adult PROP supertasters (n=31 F)	Milk (3.5% fat)	Sucrose	2%, 4%, 8%, 16%, 32% (wt/wt)	5	9-point hedonic preference scale (1 – dislike extremely; 9 – like extremely)	NSD
				Half-and-half (10.5% fat)	Sucrose	2%, 4%, 8%, 16%, 32% (wt/wt)	5		
				Heavy cream (30% fat)	Sucrose	2%, 4%, 8%, 16%, 32% (wt/wt)	5		
Drewnowski et al. (1997, 1999) [23,24]	Adult PROP non-tasters (n=43 F)	Adult PROP regular tasters (n=70 F)	Adult PROP supertasters (n=46 F)	Water	Sucrose	2%, 4%, 8%, 16%, 32% (wt/vol)	5	9-point hedonic preference scale (1 – dislike extremely; 9 – like extremely)	NSD
Mennella et al. (2005) [70]	AA; homozygous for the bitter-insensitive allele (n=45 M+F children and 34 mothers)	AP; heterozygous for the bitter-insensitive allele (n=68 M+F children and 65 mothers)	PP; homozygous for the bitter-sensitive allele (n=30 M+F children and 15 mothers)	Water	Sucrose	*3%, 6%, 12%, 24%, 36% (wt/vol)* [3, 6, 12, 24, and 36 g/100 mL]	5	Forced-choice procedure for sucrose preferences	↑ (SS) in AP and PP children *v.* AA children; in the mothers, there was no relationship between TAS2R38 genotype and sweet taste preference.

Table S3-4. Studies in which genetic/heritable factors were assessed as sweetness preference determinants (n=9).

Reference	Study Population (Sample Size)				Food Delivery Matrix	Sweetener	Sweetness		Method of Assessment	Results
							Levels	Number of Levels		
Nagy et al. (2014) [68]	PROP non-tasters		PROP supertasters		Water	Sucrose	*5.2%, 34.2% (wt/vol)*<br>[0.15, 1.0 M]	2	General labelled magnitude scale (dislike; like)	NSD within or across age groups
	<40 y (n=20 F)	>60 y (n=20 F)	<40 y (n=20 F)	>60 y (n=20 F)						
Yeomans et al. (2007) [69]	Adult PROP non-tasters (n=17 M+F)	Adult PROP medium tasters (n=25 M+F)	Adult PROP supertasters (n=18 M+F)		Water	Sucrose	*1.7%, 7.2%, 14.4%, 28.4% (wt/vol)*<br>[0.05, 0.21, 0.42, 0.83 M]	4	VAS and general LMS rating hedonic response ratings, 100-mm lines	↓ (SS) of the highest sucrose concentration in PROP supertasters *v.* non- and medium tasters

↑ = increased sweetness preference; ↓ = decreased sweetness preference; F = females; LMS = labeled magnitude scale; M = males; n = number; NR = not reported; NSD = no significant differences; PROP = 6-n-propylthiouracil; SS = statistically significant; *v.* = versus; VAS = visual analogue scale; vol = volume; wt = weight; y = years.

[a] It was suggested from the results of a genome-wide linkage analysis that the genes that affect sweetness preference may be located on chromosome 16p11.2.

Table S3-5. Studies in which body weight status was assessed as a sweetness preference determinant (n=11).

Reference	Study Population (Sample Size)		Food Delivery Matrix	Sweetener	Sweetness		Method of Assessment	Results in OW/OB *v.* NW
	NW	OW/OB			Levels	Number of Levels		
**Studies Conducted in Adults (n=8)**								
Von Atzingen and Machado Pinto e Silva (2012) [76]	NW adults (n=63 M+F)	OW+OB adults (n=60 M+F)	Orange juice	Sucrose	1%, 3%, 5%, 7%, 9% (wt/wt)	5	Pleasantness rating (1=dislike extremely; 7=like extremely)	NSD
Connolly et al. (2013) [74]	NW adults (n=10 F)	OB adults (n=10 F)	Diet Ocean Spray Cranberry Juice (<10 kcal)	Truvia	10 tsp per 10 oz	1	Taste rating (10-pt VAS)	SS ↓, despite SS greater engagement of brain networks in OB *v.* NW, as assessed using MRI
			Ocean Spray Cranberry Juice	Sucrose	*16.8% (wt/vol)* [10 tsp per 10 oz]	1	Taste rating (10-pt VAS)	SS ↓, despite SS greater engagement of brain networks in OB *v.* NW, as assessed using MRI
Ettinger et al. (2012) [72]	NW adults (n=50 F)	OW+OB adults (n=21 F)	Custard	Sugar	5%, 10%, 15%, 20% (wt/vol)	4	Pleasantness rating (1=dislike extremely; 9=like extremely)	NSD
Hardikar et al. (2017) [78]	NW adults (n=31 M+F)	OB adults (n=23 M+F)	Water	Sucrose	*10 to 40% (wt/vol)* [10.02 and 39.91 g/100 mL]	12	Pleasantness rating (-50=extremely unpleasant; +50=extremely pleasant)	NSD

Table S3-5. Studies in which body weight status was assessed as a sweetness preference determinant (n=11).

Reference	Study Population (Sample Size)		Food Delivery Matrix	Sweetener	Sweetness		Method of Assessment	Results in OW/OB *v.* NW
	NW	OW/OB			Levels	Number of Levels		
Malcolm et al. (1980) [71]	Adults (never-onset obesity; n=7 F)	Adults (juvenile-onset obesity; n=8 F)<br><br>Adults (adult-onset obesity; n=7 F)	Water	Sucrose	*0.2%, 0.4%, 1.0%, 2.1%, 3.1%, 5.1%, 10.3%, 17.1%, 27.4%, 34.2%* (wt/vol)<br><br>[6, 12, 30, 60, 90, 150, 300, 500, 800, and 1,000 mM]	10	Hedonic rating scale (0=unpleasant as anything ever tasted; 9=pleasant as anything ever tasted)	NSD
Pepino and Mennella (2012) [73]	NW adults (n=32 F)	OB adults (n=22 F)	Water	Sucrose	3%, 6%, 12%, 24%, 36% (wt/vol)	5	Monell two-series, forced-choice, paired-comparison tracking: (asked which of the two sucrose solutions they preferred)	NSD
Pepino et al. (2016) [77]	NW adults (n=19 M+F)	OB adults (n=22 M+F)	Water	Sucrose	0%, 3%, 12%, 36% (wt/vol)	4	Monell two-series, forced-choice, paired-comparison tracking: (asked which of the two sucrose solutions they preferred)	NSD

Table S3-5. Studies in which body weight status was assessed as a sweetness preference determinant (n=11).

Reference	Study Population (Sample Size)		Food Delivery Matrix	Sweetener	Sweetness		Method of Assessment	Results in OW/OB *v.* NW
	NW	OW/OB			Levels	Number of Levels		
Wooley et al. (1972) [75]	NW (n=11 M+F)	OB (n=10 M+F)	Water	Sucrose	2.5%, 5%, 10%, 20%, 40% (wt/vol)	5	Pleasantness rating (-2=very unpleasant; +2=very pleasant), before and 1 hr after ingestion of 200 mL of a 25% glucose solution	NSD
							Pleasantness rating (-2=very unpleasant; +2=very pleasant), before and 1 hr after ingestion of 200 mL of an isosweet cyclamate solution	NSD
**Studies Conducted in Infants or Children (n=3)**								
Alexy et al. (2011) [79]	NW children (n=426; GD NR)	OW+OB children (n=148; GD NR)	Apple juice	Sucrose	0.4%, 1.0% (wt/vol)	2	Series of paired comparisons (asked "Which food do you like more?")	NSD
Lanfer et al. (2012) [81]	NW Children (n=1,300 M+F)	OW+OB children (n=396 M+F)	Apple juice	Sucrose	0.53%, 3.11% (wt/vol)	2	Series of paired comparisons (asked "Which food do you like more?")	↑ for 3.11% (OR for OW or OB in individuals preferring the sweeter apple juice was 1.5 (95% CI: 1.1, 2.1)[a]

Table S3-5. Studies in which body weight status was assessed as a sweetness preference determinant (n=11).

Reference	Study Population (Sample Size)		Food Delivery Matrix	Sweetener	Sweetness		Method of Assessment	Results in OW/OB *v.* NW
	NW	OW/OB			Levels	Number of Levels		
Grinker et al. (1986) [80]	Infants from non-OB mothers (n=20 M+F)	Infants from OB mothers (n=20 M+F)	Water	Sucrose	*2.1%, 4.3%, 8.6%, 20.5% (wt/vol)* [0.0612, 0.125, 0.25, 0.6M]	4	Total number of sucks, number of bouts of sucking, rate, and amplitude of the responses to each solution were analyzed	NSD

BMI = body mass index; CI = confidence intervals; F = females; GD = gender distribution; hr = hour; M = males; MRI = magnetic resonance imaging; n = number; NSD = no significant differences; NW = normal weight; OB = obese; OR = odds ratio; OW = overweight; pt = point; SS = statistically significant; tsp = teaspoons; VAS = visual analogue scale; vol = volume; wt = weight

[a] Adjusted for age, sex, survey center, parental education, BMI of father and BMI of mother.

Table S3-6. Studies in which weight loss was assessed as a sweetness preference determinant (n=5).

Reference<br><br>Study Design	Study Population (Sample Size)	Food Delivery Matrix	Sweetener	Sweetness		Method of Assessment	Results
				Levels	Number of Levels		
Alexy et al. (2010) [82]<br><br>Before-and-after	Obese children and adolescents (n=72 M+F) undergoing a 12-mo outpatient weight loss intervention	Apple juice	Sucrose	*0.4%, 1.0% (wt/vol)*<br><br>[4, 10 g/L]	2	Paired comparison preference tests, assessed with the question, "*Which food do you like more?*"	NSD
Asao et al. (2016) [83]<br><br>Before-and-after	Obese adults (20 M+F) consuming a VLCD as part of a 6-mo medical weight management program	Water	Sucrose	*1.5%, 2.3%, 3.3%, 4.9%, 7.3%, 10.6%, 15.4%, 22.1%, 31.0%, 42.4% (wt/vol)*<br><br>[0.043, 0.066, 0.097, 0.144, 0.212, 0.310, 0.450, 0.645, 0.905, and 1.239 M]	10	Forced-choice, paired-comparison tracking method	NSD
Burgess et al. (2016) [84]<br><br>R, P	Obese Adults (n=31 F) consuming a LFD for 6 mo<br><br>Obese adults (n=38 F) consuming a LCD for 6 mo	Strawberry milk	Sucrose	0%, 15%, 30% (wt/vol)	3	Overall Liking Rating (0cm=not at all; 15cm=very much)	At baseline, ↑ (SS) for 15% and 30% sucrose over 0% sucrose; however, at 6 months ↑ (SS) for 15% sucrose over 0% and 30% sucrose.

Table S3-6. Studies in which weight loss was assessed as a sweetness preference determinant (n=5).

Reference<br><br>Study Design	Study Population (Sample Size)		Food Delivery Matrix	Sweetener	Sweetness		Method of Assessment	Results
					Levels	Number of Levels		
Drewnowski and Holden-Wiltse (1992) [85]<br><br>P	Obese adolescents and adults with high weight fluctuations (n=17 F)	Obese adolescents and adults with low weight fluctuations (n=20 F)	Water	Sucrose	0, 2, 8, 16, 32% (wt/vol)	5	9-point hedonic preference scale (dislike extremely; like extremely)	↑ (SS) in subjects with high *v.* low weight fluctuations for ice cream but not sucrose solutions.
			Ice cream	Sucrose	12, 15, 18% (wt/wt)	3		
Kleifield and Lowe (1991) [86]<br><br>P	Adults with reported recent weight loss (high recent weight loss: n=10 F; low recent weight loss: n=10 F)	Adults with reported past weight loss (high past weight loss: n=10 F; low past weight loss: n=10 F)	Water	Sucrose	2.5%, 5%, 10%, 20%, 40% (wt/vol)	5	5-point hedonic preference scale (-2 – very unpleasant; +2 very pleasant) (pre-glucose load)<br>5-point hedonic preference scale (-2 – very unpleasant; +2 very pleasant) (post-glucose (25% [wt/v]) load)	↓ (SS) in those with high past weight loss *v.* those with low past weight loss (both before and after the glucose load). ↑ (SS) in those with high recent weight loss *v.* those with low recent weight loss (following but not prior to the glucose load).

↑ = increased sweetness preference; ↓ = decreased sweetness preference; F = females; LCD = low carbohydrate diet; LFD = low fat diet; M = males; mo = months; n = number; NSD = no significant differences; SS = statistically significant; VLCD = very low calorie diet; vol = volume; wt = weight

Table S3-7. Studies in which sound was assessed as a sweetness preference determinant (n=2).

Reference<br><br>Study Design	Study Population (Sample Size)[a] Control	Study Population (Sample Size)[a] Comparator(s)	Food Delivery Matrix	Sweetener	Sweetness Levels	Sweetness Number of Levels	Method of Assessment	Results
Ferber and Cabanac (1987) [87]<br><br>R, X	Silence (Adults; n=10 M)	Low noise, 70 dB<br><br>Loud noise, 90 dB<br><br>Loud music, 90 dB	Water	Sucrose	*5.1%, 10.3%, 19.9%, 40.0%, 80.4%* (wt/vol) [0.15, 0.30, 0.58, 1.17, 2.35 M]	5	Magnitude estimation scale without anchor (positive – pleasure; negative – displeasure)	🡅 at 2.1% (SS): loud noise *v.* control; 🡅 at 8.3% (SS): loud noise and loud music *v.* control; 🡅 for mean (SS): loud noise and music *v.* control
Kontukoski et al. (2015) [29]<br><br>R, P	"Sweet" music: Satie (Adults; n=18 M+F) and Schuman (Adults; n=12 M+F)	"Sour" music: Ferneyhough (Adults; n=20 M+F) and Mesz (Adults; n=15 M+F)	Mango juice	Sugar	10% (wt/vol)	1	Beverage preparation (self-selection of ingredients while listening to music)	↑ use of sweet ingredients (SS): "sweet" *v.* "sour" music
			Orange juice	Sugar	10% (wt/vol)	1		
			Grapefruit juice	Sugar	9% (wt/vol)	1		
			Lemon juice	Sugar	1.6% (wt/vol)	1		
			Pineapple juice	Sugar	30% (wt/vol)	1		
			Liquid honey	Sugar	80% (wt/vol)	1		

🡅 = increased sweetness preference; M = males; n = number; P = parallel; R = randomized; SS = statistically significant; *v.* = versus; vol = volume; wt = weight; X = crossover.

[a] Sample sizes are reported for the comparator(s) only if the study was parallel in design.

Table S3-8. Studies in which personality traits were assessed as sweetness preference determinants (n=5).

Reference	Study Populations (Sample Size)	Food Delivery Matrix	Sweetener	Sweetness		Method of Assessment	Observation in Control vs. Test
				Levels	Number of Levels		
Kampov-Polevov et al. (2006) [88]	Adults (n=163 M+F)	Water	Sucrose	*1.7%, 3.4%, 7.2%, 14.4%, 28.4% (wt/vol)* [0.05, 0.10, 0.21, 0.42, 0.83 M]	5	Sweet Taste Questionnaire, 7-point Likert scale (1 – strongly disagree; 7 – strongly agree); 200-mm analog scale ("How much do you like the taste?")	Liking of the strongest sucrose solution was associated with SS greater scores for the mood-altering effect of sweets and impaired control over-eating sweets.
Saliba et al. (2009) [89]	Adults (n=45 M+F)	White wine	Fructose	*0%, 2% (wt/vol)* [0, 20 g/L]	2	Wine preference test	↑ was SS associated with greater impulsiveness and lower openness
Sena-Esteves et al. (2018) [90]	Adults (n=114 M+F)	Red wine	Glucose and fructose (1:1)	*0.2%, 0.4%, 0.8%, 1.6%, 3.2% (wt/vol)* [2, 4, 8, 16, 32 g/L]	5	Two ascending forced choice protocol (paired comparisons)	SS ↑ in F *v.* M<br><br>SS ↑ in novices *v.* experienced alcohol drinkers<br><br>SS ↑ for 8 g/L in low *v.* high extroverts
Stone and Pangborn (1990) [92]	Adults (n=100 M+F)	Lemonade	Sucrose	4%, 6%, 8%, 10%, 14%, 20%, 30% (wt/vol)	7	Hedonic rating, 20-point scale (dislike; like)	SS ↑ in highly outgoing *v.* medium or low outgoing individuals<br><br>SS ↑ in subjects with a low or medium *v.* high Type A personality

Table S3-8. Studies in which personality traits were assessed as sweetness preference determinants (n=5).

Reference	Study Populations (Sample Size)	Food Delivery Matrix	Sweetener	Sweetness		Method of Assessment	Observation in Control vs. Test
				Levels	Number of Levels		
Weafer et al. (2014) [91]	Adults (n=95 M+F)	Cherry Kool-Aid	Sucrose	*1.7%, 3.4%, 7.2%, 14.4%, 28.4% (wt/vol)* [0.05, 0.10, 0.21, 0.42, 0.83 M]	5	Hedonic rating, 100-mm VAS (disliked very much; liked very much)	SS ↑ in subjects with greater *v.* less impulsive choice<br><br>NSD in sweetness preference between subjects based on impulsive action

↑ = increased sweetness preference; ↓ = decreased sweetness preference; b/w = between; F = females; M = males; n = number; NSD = no significant differences; SS = statistically significant; *v.* = versus; VAS = visual analoge scale; vol = volume; wt = weight

Table S3-9. Studies in which ethnicity and lifestyle were assessed as sweetness preference determinants (n=10).

Reference	Study Population (Sample size)			Food Delivery Matrix	Sweetener	Sweetness		Method of Assessment	Results
	Population 1	Population 2	Population 3			Levels	Number of Levels		
**Studies in Which Different Ethnic Groups were Investigated (n=5)**									
Bertino et al. (1983) [93]	University of Pennsylvania students of European descent, born in U.S. (n=34 M+F)	University of Michigan students of Chinese descent, born in Taiwan (n=28 M+F)	NA	Water	Sucrose	*2.1%, 3.8%, 6.5%, 11.6%, 20.2%, 34.2%, 61.6% (wt/vol)* [0.06, 0.11, 0.19, 0.34, 0.59, 1.0, 1.8 M]	7	Pleasantness rating on a 9-point hedonic scale (1 – very bad; 9 – very good)	NSD b/w groups in the breakpoint sucrose concentration<br><br>↑ in subjects of Chinese descent (p=0.06)
	University of Pennsylvania students of European descent, born in U.S. (subset of above study group; n=22 M+F)	University of Michigan students of Chinese descent, born in Taiwan (subset of above study group; n=10 M+F)	NA	Cookies	Sucrose	8, 14, 20, 25, 30, 34, and 37% (wt/wt, wet)	7	Pleasantness rating on a 9-point hedonic scale (1 – very bad; 9 – very good)	NSD b/w groups in the breakpoint sucrose concentration<br><br>SS sucrose by group interaction such that the students of Chinese descent rated the least sweet cookies as tasting most pleasant

Table S3-9. Studies in which ethnicity and lifestyle were assessed as sweetness preference determinants (n=10).

Reference	Study Population (Sample size)			Food Delivery Matrix	Sweetener	Sweetness		Method of Assessment	Results
	Population 1	Population 2	Population 3			Levels	Number of Levels		
Holt et al. (2000) [95]	Australian-born Caucasian university students living in Australia (n=69 M+F)	Malaysian-born [mostly (73%) of Malay descent] university students living in Australia (n=63 M+F)	NA	Water	Sucrose	2%, 4%, 8%, 16%, 32% (wt/vol)	5	Liking ratings on a 150-mm line scale with the opposing extremes, "dislike extremely" and "like extremely"<br><br>Differences b/w groups in the most preferred sucrose levels, the mean sucrose solution rating, and ratings at each sucrose level were assessed[a]<br><br>Subjects also completed an FFQ, which was designed to assess habitual intakes of sugar, artificial sweeteners, and sweet foods and drinks[a]	SS ↑ for the 8% solution and SS ↓ for the 32% solution in Malaysians *v.* Australians<br><br>SS +ve correlation b/w the most preferred sucrose level and the frequency of consumption of sweet foods and drinks and total and refined sugars
				Orange juice	Sucrose	0%, 5%, 10%, 20% (wt/vol)	5		The mean sucrose rating was SS greater in Malaysians *v.* Australians
				Custard	Sucrose	0%, 5%, 10%, 20% (wt/vol)	5		SS ↓ for the 0% and 5% custards in. Malaysians *v* Australians.<br>The most preferred sucrose level was SS greater in Malaysians *v.* Australians
				Shortbread biscuit	Sucrose	0%, 5%, 10%, 20% (wt/vol)	5		The mean sucrose rating was SS greater in Australians *v.* Malaysians

Table S3-9. Studies in which ethnicity and lifestyle were assessed as sweetness preference determinants (n=10).

Reference	Study Population (Sample size)			Food Delivery Matrix	Sweetener	Sweetness		Method of Assessment	Results
	Population 1	Population 2	Population 3			Levels	Number of Levels		
Jaafar and Abdul Razak (1990) [94]	Malay children living in Malaysia (n=60 M+F)	Chinese children living in Malaysia (n=60 M+F)	Indian children living in Malaysia (n=40 M+F)	Water	Sucrose	*0%, 10.3%, 20.5%, 30.8%, 41.1% (wt/vol)* [0.0, 0.3, 0.6, 0.9, 1.2 M]	5	Preference test (full-mouth technique)	SS ↑ in Malay and Indian *v.* Chinese children
Takemi and Woo (2017) [96]	Korean children (n=95 M+F)	Japanese children (n=71 M+F)	NA	Banana milk	Sugar	1.7%, 3.5%, 7%, 14%, 28% (wt/vol)	5	Sweetness preference test (modified paired-comparison test)	NSD
Tuorila et al. (2017) [97]	British adults (n=987 M+F)	Finnish adults (n=468 M+F)	NA	Water	Sucrose	20% (wt/vol)	1	Hedonic degree of liking, 120-mm LAM scale (-60 – greatest imaginable dislike; +120 – strongest imaginable sensation)	NSD

Table S3-9. Studies in which ethnicity and lifestyle were assessed as sweetness preference determinants (n=10).

Reference	Study Population (Sample size)			Food Delivery Matrix	Sweetener	Sweetness		Method of Assessment	Results
	Population 1	Population 2	Population 3			Levels	Number of Levels		
**Studies in Individuals with Traditional versus Modern Lifestyles (n=3)**									
Jamel et al. (1996) [98]	Urban 1, children and adults from families where the father completed higher education (n=1,203 M+F)<br><br>Urban 2, children and adults from families where the father completed lower education (n=1, 352 M+F)	Rural 1, children and adults from families who lived <200 km from Baghdad (n=798 M+F)<br><br>Rural 2, children and adults from families who lived >200 km away from Baghdad (800 M+F)	NA	Tea	Sucrose	*0%, 5.1%, 9.9%, 15.1%, 20.2% (wt/vol)* [0, 0.15, 0.29, 0.44, 0.59 M]	5	Sweet Preference Inventory	SS ↑ for 0.59 M in urban *v.* rural subjects.<br><br>SS ↓ for 0.59 M in Urban 1 *v.* Urban 2 subjects.<br><br>NSD b/w Rural 1 and Rural 2 subjects in preference for 0.59 M.

Table S3-9. Studies in which ethnicity and lifestyle were assessed as sweetness preference determinants (n=10).

Reference	Study Population (Sample size)			Food Delivery Matrix	Sweetener	Sweetness		Method of Assessment	Results
	Population 1	Population 2	Population 3			Levels	Number of Levels		
Salbe et al. (2004) [99]	Pima Indian adults (n=123 M+F)	White adults (n=65 M+F)	NA	Non-fat milk (0.1% fat)	Sugar	0%, 5%, 10%, 20% (wt/wt)	4	Hedonic response rating of pleasantness, 100-mm VAS	SS ↑ in white adults *v.* Pima Indians.
				Whole milk (3.5% fat)	Sugar	0%, 5%, 10%, 20% (wt/wt)	4		
				Half and half (11.3% fat)	Sugar	0%, 5%, 10%, 20% (wt/wt)	4		
				Cream (37.5% fat)	Sugar	0%, 5%, 10%, 20% (wt/wt)	4		
Sorokowska et al. (2017) [100]	Tanzania (Hadza, hunger-gatherers; n=85 M+F)	Amazon/ Bolivia (Tsimane', forager-horti-culturalists; n=138 M+F)	Modern society (Poles; n=200 M+F)	Water	D-saccharose	10% (wt/vol) [10 g/100 mL]	1	Hedonic response rating (I like it a lot; I do not like it at all)	SS ↓ in Hadza *v.* Tsimane' and Poles
**Studies in Which Subjects with Different Levels of Physical Activity were Investigated (n=2)**									
Crystal et al. (1995) [102]	Adult non-athletes (fall controls; n=17 F)	Adult non-athletes (winter controls; n=11 F)	Adult competitive swimmers (n=16 F)	Skim milk (0% fat)	Sucrose	0%, 5%, 10%, 20% (wt/wt)	4	Hedonic response rating of pleasantness, 160-mm analog rating scale (extremely unpleasant; extremely pleasant)	SS ↓ for 20% in swimmers *v.* non-athletes; SS ↑ for 0% and 10% in swimmers *v.* non-athletes
				Whole milk (3.5% fat)	Sucrose	0%, 5%, 10%, 20% (wt/wt)	4		
				Half and half (10.5% fat)	Sucrose	0%, 5%, 10%, 20% (wt/wt)	4		
				Heavy cream (37.6% fat)	Sucrose	0%, 5%, 10%, 20% (wt/wt)	4		

Table S3-9. Studies in which ethnicity and lifestyle were assessed as sweetness preference determinants (n=10).

Reference	Study Population (Sample size)			Food Delivery Matrix	Sweetener	Sweetness		Method of Assessment	Results
	Population 1	Population 2	Population 3			Levels	Number of Levels		
Martin and Bellisle (1989) [101]	Sedentary health adult controls (n=14 F)	Young ballerina adults (n=23 F)	NA	Soft-white cheese (0% fat wt/wt)	Sucrose	1%, 5%, 10%, 20%, 40% (wt/wt)	5	Hedonic sweetness preference, 9-point category scale (extremely unpleasant; extremely pleasant)	The "interpolated" optimally preferred sucrose level in dancers was lower than in controls (SS NR).
				Soft-white cheese (20% fat wt/wt)	Sucrose	1%, 5%, 10%, 20%, 40% (wt/wt)	5		
				Soft-white cheese (40% fat wt/wt)	Sucrose	1%, 5%, 10%, 20%, 40% (wt/wt)	5		
				Heavy cream (30% fat wt/wt)	Sucrose	1%, 5%, 10%, 20%, 40% (wt/wt)	5		

🡡 = increased sweetness preference; 🡣 = decreased sweetness preference; +ve = positive; b/w = between; F = females; FFQ = food frequency questionnaire; LAM = labeled affective magnitude; M = males; n = number; NA = not applicable; NR = not reported; NSD = no significant differences; SS = statistically significant; U.S. = United States; *v.* = versus; VAS = visual analogue scale; vol = volume; wt = weight

[a] Given that several outcomes were assessed, only those found to be SS different b/w groups are summarized under the "Results" column.

Table S3-10. Studies in which previous exposure to sweets was assessed as a sweetness preference determinant (n=6).

Reference<br><br>Study Design	Study Population (Sample Size)		Food Delivery Matrix	Sweetener	Sweetness		Method of Assessment	Results
	Previous Exposure to Sweet	Previous Exposure to Non-Sweet			Levels	Number of Levels		
**Studies Conducted in Children Considering Previous Exposure to Sweet Foods (n=4)**								
Brown and Grunfeld (1980) [58]<br><br>P	Infants fed baby foods with added sugars for the first 3 mo (n=15 M+F)	Infants with no added sugar intakes for the first 3 mo (n=15 M+F)	Baby food	Sugar	Unsweetened, sweetened	2	Parent's perception of baby's preference on 5-point scale (1-low; 5-high) during a 4-week period	NSD
Liem and Mennella (2002) [105]	Children 4-5 y fed milk-based formula during infancy (n=21 M+F)<br><br>Children 6-7 y fed milk-based formula during infancy (n=27 M+F)	Children 4-5 y fed hydrolysate formula during infancy (n=18 M+F)<br><br>Children 6-7 y fed hydrolysate formula during infancy (n=17 M+F)	Apple juice	Sucrose	*5.5%, 7.5%, 16.1%, 22.6%, 31.8% (wt/vol)* [0.16, 0.22, 0.47, 0.66, 0.93 M]	5	Forced-choice procedure (most preferred of paired solutions)	NSD based on previous infant formula<br><br>SS 🡩 in children whose mothers reported adding sugar to their foods
Liem *et al*. (2004) [106]<br><br>P	Children with parents who exert low restriction over sugary foods (n=21 M+F)	Children with parents who exert high restriction over sugary foods (n=22 M+F)	Orangeade	Sucrose	*4.8%, 6.8%, 9.9%, 14.4%, 20.9%* (wt/vol) [0.14, 0.20, 0.29, 0.42, 0.61 M]	5	Paired-comparison test ("which one do you like best?" for each pair) and rank-order test (most preferred; least preferred)	SS 🡩 in children with high *v.* low restriction

Table S3-10. Studies in which previous exposure to sweets was assessed as a sweetness preference determinant (n=6).

Reference<br><br>Study Design	Study Population (Sample Size)		Food Delivery Matrix	Sweetener	Sweetness		Method of Assessment	Results
	Previous Exposure to Sweet	Previous Exposure to Non-Sweet			Levels	Number of Levels		
Fry Vennerød et al. (2017) [107]<br><br>P	Children (n=135 M+F)		Fruit-flavoured beverage, chocolate	Sugar	4%, 12%, 18% (wt/vol)	4	Rank-order test by elimination (most preferred; least preferred)	SS ↑ in children with more frequent exposure to sweet foods and snacks
**Studies Conducted in Children Considering Previous Exposure to Sweetened Water (n=2)**								
Beauchamp and Moran (1982) [103]<br><br>P	Infants exposed to sweetened water in the first 6 mo (black: n=28 M+F; white: n=8 M+F)	Infants not exposed to sweetened water in the first 6 mo (black: n=74 M+F; white: n=21 M+F)	Water	Sucrose	*0%, 6.8%, 20.5% (wt/vol)* [0, 0.2, 0.6 M]	3	Intakes of test solutions	SS ↑ at 6 mo in infants fed sweetened water *v.* infants not fed sweetened water.
	Infants exposed to sweetened water at 6 mo (black: n=28 M+F; white: n=8 M+F)	Infants not exposed to sweetened water at 6 mo (black: n=74 M+F; white: n=21 M+F)	Water	Sucrose	*0%, 6.8%, 20.5% (wt/vol)* [0, 0.2, 0.6 M]	3		

Table S3-10. Studies in which previous exposure to sweets was assessed as a sweetness preference determinant (n=6).

Reference<br><br>Study Design	Study Population (Sample Size)		Food Delivery Matrix	Sweetener	Sweetness		Method of Assessment	Results
	**Previous Exposure to Sweet**	**Previous Exposure to Non-Sweet**			**Levels**	**Number of Levels**		
Beauchamp and Moran (1984) [104]<br><br>P	2-y-old children fed sweetened water for up to the first 6 mo (n=18 M+F)<br><br>2-y-old children fed sweetened water for greater than the first 6 mo (n=29 M+F)	2-y-old children never fed sweetened water (n=16 M+F)	Water, Kool-Aid	Sucrose	*0%, 6.8%, 20.5% (wt/vol)* [0, 0.2, 0.6 M]	3	Intakes of test solutions	SS ↑ at 2 y in both groups of infants previously fed sweetened water<br><br>NSD in preference for Kool-Aid<br><br>SS ↑ in children for whom prior ingestion of Kool-Aid was reported

↑ = increased sweetness preference; ↓ = decreased sweetness preference; F = females; M = males; mo – months; n = number; NSD = no significant differences; P = parallel; SS = statistically significant; *v.* = versus; vol = volume; wt = weight; y = years

Table S3-11. Studies in which disease was assessed as a sweetness preference determinant (n=12).

Reference	Study Population		Food Delivery Matrix	Sweetener	Sweetness		Method of Assessment	Observation in Patients With *v.* Without Disease
	Control (sample size)	Disease (Sample Size)			Levels	Number of Levels		
**Studies in Patients with a Neurological/Psychological Disorder (n=7)**								
Berlin et al. (1998) [111]	Adults (healthy; n=20 M+F)	Adults (major depression; n=20 M+F)	Water	Sucrose	0%, 5%, 10%, 20%, 40% (wt/wt)	5	Pleasantness rating (1=extremely unpleasant; 9=extremely pleasant)	NSD
Franko et al. (1994) [110]	Adults (healthy; n=20 F)	Adults (with narrowly defined BN; n=15 F)<br><br>Adults (with BN and a history of AN; n=5 F)	Water	Sucrose	0%, 1.3%, 2.6%, 5%, 10%, 20%, 40% (wt/vol)	7	Pleasantness rating (0mm=extremely unpleasant; 100mm=extremely pleasant)	SS ↑ for 40%: Patients with narrowly defined BN *v.* control & patients with a history of AN
Goodman et al. (2018) [114]	Adults (binge-eating disorder; n=26)		Water	Sucrose	*1.7, 3.4, 7.2, 14.4, 28.4% (wt/vol)*<br><br>[0.05, 0.10, 0.21, 0.42, 0.83 M]	5	200mm pleasantness VAS (Disliked very much; Liked very much – specific values NR)	SS ↑ frequency of binge-eating and over-eating frequencies in the past 28 days: patients with the highest *v.* lower preference for sweets

Table S3-11. Studies in which disease was assessed as a sweetness preference determinant (n=12).

Reference	Study Population		Food Delivery Matrix	Sweetener	Sweetness		Method of Assessment	Observation in Patients With *v.* Without Disease
	Control (sample size)	Disease (Sample Size)			Levels	Number of Levels		
**Studies in Patients with Neurological or Psychological Disease (n=7)**								
Sunday and Halmi (1990) [108]	Adults (healthy; n=26 GD NR)	Adults (normal weight bulimics; n=42 GD NR)	Water	Sucrose	0%, 1.78%, 3.16%, 5.62%, 10%, 17.78%, 31.62% (wt/wt)	7	Pleasantness rating (1=dislike extremely; 9=like extremely)	SS ↓ for 1.78% & 3.16%: Anorexic restrictors *v.* controls
		Adults (anorexic bulimics; n=36 GD NR)<br><br>Adults (anorexic-restrictors; n=48 GD NR)	Dairy solution	Sucrose	0%, 5%, 10%, 20% (wt/wt)	4	Pleasantness rating (1=dislike extremely; 9=like extremely)	SS ↓ for 0%: Anorexic restrictors *v.* controls
Sienkiewicz-Jarosz et al. (2013) [112]	Adults (healthy; n=20 M+F)	Adults (PD; n=20 M+F)	Water	Sucrose	0%, 1%, 10%, 30% (wt/wt)	4	Pleasantness rating (-50mm=extremely unpleasant; 50mm=extremely pleasant)	NSD
Swiecicki et al. (2015) [113]	Adults (non-depressed; n=30 M+F)<br><br>Adults (depressed but non-SAD; n=20 M+F)	Adults (depressed with SAD; n=18 M+F)	Water	Sucrose	0%, 1%, 10%, 30% (wt/wt)	4	Pleasantness rating (-50mm=extremely unpleasant; 50mm=extremely pleasant)	NSD

Table S3-11. Studies in which disease was assessed as a sweetness preference determinant (n=12).

Reference	Study Population		Food Delivery Matrix	Sweetener	Sweetness		Method of Assessment	Observation in Patients With *v.* Without Disease
	Control (sample size)	Disease (Sample Size)			Levels	Number of Levels		
Travers et al. (1993) [109]	Adults (n=16 M+F)	Adults (PD; n=25 M+F)	Water	Sucrose	*2.7, 5.1, 10.3, 30.8, 51.3%* (wt/vol)<br><br>[0.08M, 0.15M, 0.3M, 0.6M, 0.9M, 1.5M]	6	Preference rating (1=extremely like; 6=extremely dislike)	In an ANOVA, there was a SS concentration effect (P=0.003) and disease X concentration effect (P<0.001), such that at higher sucrose concentrations, PD patients preferred the increased sweetness compared to dislike from controls
**Studies in Patients with T2DM or GDM (n=3)**								
Tepper et al. (1996) [116]	Adults (non-diabetic; n=16 M+F)	Adults (T2DM; n=21 M+F)	Cherry-flavored beverage	Sucrose	1.5%, 3%, 6%, 12%, 24% (wt/wt)	5	Pleasantness rating (0cm=extremely unpleasant; 15cm=extremely pleasant)	NSD
			Cherry-flavored beverage	Fructose	1%, 2%, 5%, 10.2%, 18% (wt/wt)	5	Pleasantness rating (0cm=extremely unpleasant; 15cm=extremely pleasant)	NSD
			Cherry-flavored beverage	Aspartame	0.25%, 0.5%, 1%, 2%, 4% (wt/wt)	5	Pleasantness rating (0cm=extremely unpleasant; 15cm=extremely pleasant)	NSD

Table S3-11. Studies in which disease was assessed as a sweetness preference determinant (n=12).

Reference	Study Population		Food Delivery Matrix	Sweetener	Sweetness		Method of Assessment	Observation in Patients With *v.* Without Disease
	Control (sample size)	Disease (Sample Size)			Levels	Number of Levels		
Tepper and Seldner (1999) [115]	Non-pregnant (n=12 F)<br><br>Adults (pregnant without GDM; n=30 F)	Pregnant with GDM (n=25 F)	Strawberry-flavored milk	Sucrose	0%, 5%, 10% (wt/vol)	3	Pleasantness rating (1= dislike extremely; 9=like extremely)	SS ↑ for 10% in pregnant women with GDM *v.* pregnant women without GDM.
			Water	Glucose	*0.3 to 5.5%*<br><br>[10-160 mmol/L]	5	Pleasantness rating (0cm=dislike extremely; 15cm=like extremely)	NSD
Yu et al. (2014) [117]	Adults (without T2DM; n=100 M+F)	Adults (with T2DM; n=100 M+F)	Water	Sucrose	*3.1, 6.2, 12, 24, 36% (wt/vol)*<br><br>[90, 180, 350, 700, 1,050 mM]	5	Series of paired comparisons: ("which one do you prefer?" for each pair)	SS ↓ in T2DM *v.* non-T2DM
**Studies in Patients with Other Diseases (n=2)**								
Bellisle et al. (1990) [118]	Children (healthy; n=25 M+F)	Children (end-stage renal disease; n=39 M+F)	Soft white cheese	Sucrose	1%, 5%, 10%, 15%, 20% (wt/wt)	5	Series of paired comparisons: (asked which of the two adjacent sucrose concentrations in the same test food they preferred)	↓ for 20% solution (SS): End-stage renal disease *v.* healthy<br><br>↑ for 1% solution (SS): End-stage renal disease *v.* healthy
			Apple sauce	Sucrose	10%, 20%, 30%, 40%, 60% (wt/wt)	5	Series of paired comparisons (asked which of the two adjacent sucrose concentrations in the same test food they preferred)	Similar preference trends were observed for apple sauce as were observed for soft white cheese

Table S3-11. Studies in which disease was assessed as a sweetness preference determinant (n=12).

Reference	Study Population		Food Delivery Matrix	Sweetener	Sweetness		Method of Assessment	Observation in Patients With *v.* Without Disease
	Control (sample size)	Disease (Sample Size)			Levels	Number of Levels		
Prince et al. (1999) [119]	Adults (Healthy; n=6 M+F)	Adults (PWS; n=9 M+F)	Water	Sucrose	0.75%, 1%, 1.25%, 1.5% (wt/wt)	4	Series of paired comparisons (asked which of the two adjacent sucrose concentrations in the same test food they preferred)	🡅 (statistics NR): PWS vs healthy, for all 3 sweeteners tested
				Fructose	0.75%, 1%, 1.25%, 1.5% (wt/wt)	4		
				Aspartame	0.75%,1%, 1.25%, 1.5% (wt/wt)	4		

🡅 = increased sweetness preference; 🡇 = decreased sweetness preference; AN = anorexia nervosa; ANOVA = analysis of variance; BN = bulimia nervosa; F = females; GD = gender distribution; GDM = gestational diabetes mellitus; M = males; n = number; NR = not reported; NSD = no significant differences; PD = Parkinson's disease; PWS = Prader-Willi syndrome; SAD = seasonal affective disorder; SS = statistically significant; T2DM = type 2 diabetes mellitus; *v.* = versus; VAS = visual analogue scale; vol = volume; wt = weight.

Table S3-12. Studies in which other factors were assessed as sweetness preference determinants (n=8).

Reference<br><br>Study Design	Study Population (Sample size)		Food Delivery Matrix	Sweetener	Sweetness		Method of Assessment	Results
					Levels	Number of Levels		
**Studies in which Various Factors were Assessed as Sweetness Preference Determinants (n=3)**								
Frijters (1984) [120]<br><br>P	Adult restrained eaters: low (n=8 F), medium (n=7 F), and high (n=8 F)		Water	Sucrose	*2.1%, 3.9%, 7.2%, 10.5%, 23.7%, 44.5% (wt/vol)* [0.06, 0.1148, 0.2089, 0.3082, 0.6918, and 1.3 (mol/L)]	6	Pleasantness rating (165mm line: middle = ideal sweetness; left = less sweet than ideal; right = more sweet than ideal)	NSD.
Conroy et al. (2014) [28]<br><br>P	Weight-stable F after RYGB, 16-wk placebo treatment (n=11 F)	Weight-stable F after RYGB, 16-wk leptin treatment (n=11 F)	Liquid meal (Optifast)	CHO	50% of energy, equivalent to 40 g	NA	Subjective feeling 100-mm VAS ("How much do you crave something sweet?"), assessed at 0, 15, 30, 60, 90, and 120 min following consumption of Optifast	SS reduction in sweet craving with leptin *v.* placebo treatment.
Habhab et al. (2009) [121]<br><br>P	Low Stress adults (n=20 F with either low or high dietary restraint)[a]	High Stress adults (n=20 F with either low or high dietary restraint)[a]	Honey-flavored Teddy Grahams (sweet, low fat)	Sucrose	9 g per 30 g serving	1	Before- and after-weights of snack foods provided	High Stress group ate SS more sweet food than did the Low Stress group.<br><br>High Stress group ate SS more high fat food than did the Low Stress group.<br><br>High Stress group ate SS more sweet, high fat foods than did the Low Stress group.
			Plain M&M chocolate candies (sweet, high fat)	Sucrose	38 g per 50 g serving	1		

Table S3-12. Studies in which other factors were assessed as sweetness preference determinants (n=8).

Reference<br><br>Study Design	Study Population (Sample size)	Food Delivery Matrix	Sweetener	Sweetness		Method of Assessment	Results
				Levels	Number of Levels		
**Studies in which Associations with Multiple Sweetness Preference Determinants were Assessed (n=5)**							
Ahrens (2015) [125]<br><br>Cross-sectional Observational	Children 6 to 9 years of age (n=1,839 M+F; GD NR)	Apple juice	Sucrose	0.53%, 3.11% added sucrose[b] (%-type NR)	2	Paired forced choice test	SS +ve associations were reported for sweetness preference and country of residence, age, and BMI.
Gawecki et al. (1976) [123]<br><br>Cross-sectional Observational	Cadets (n=83 M)	Tea drink (15 g tea blend/1 L water)	Saccharose	Low, moderate, high	3	Self-preparation of beverage to preferred level of sweetness	The following SS correlations were identified with sweet taste preference (all +ve): sweet taste sensitivity, fasting blood glucose, basal metabolic rate, and body weight index.
Jayasinghe *et al*. (2017) [124]<br><br>Cross-sectional Observational	Adults (n=44 F)	Water	Glucose	*4.3%, 8.6%, 17.1%, 34.2% (wt/vol)* [125, 250, 500, 1,000 (mM)]	4	General labelled magnitude 100-mm scale of hedonic liking (-100 – extremely dislike; +100 – extremely like)<br><br>4-day weighed food record<br><br>Sweet FFQ<br><br>Sweet beverage liking questionnaire	SS +ve correlation b/w sweetness preference and total energy and CHO (total sugar, fructose, glucose) intakes.

Table S3-12. Studies in which other factors were assessed as sweetness preference determinants (n=8).

Reference<br><br>Study Design	Study Population (Sample size)	Food Delivery Matrix	Sweetener	Sweetness		Method of Assessment	Results
				Levels	Number of Levels		
Kim *et al*. (2006) [122]<br><br>Cross-sectional Observational	Prospective nutrition teachers (n=30; GD NR)	Omija jelly	Sucrose	12%, 14%, 16%, 18% (%-type NR)	4	Asked to choose the jelly with the most optimal level of sweetness preference,	NSD between optimal level of sweetness and physical activity, stress, sleep, weight gain, constipation, dietary habits and *etc*.
Lampuré *et al*. (2015) [30]<br><br>Cross-sectional Observational	Adults (n=37,181; 8,677 M + 28,504 F)	NA	NA	NA	NA	Validated questionnaire of sensory liking (e.g., saltiness, sweetness, fatty)	SS -ve associations with sweetness preference and: age (in M and F); current smoking status (in F); stronger cognitive restraint (in M and F); former dieting (in M and F).<br><br>SS +ve associations with sweetness preference and: uncontrolled eating habits (in M and F); emotional eating habits (in M and F).

🡅 = increased sweetness preference; +ve = positive; -ve = negative; CHO = carbohydrate; F = females; FFQ = food frequency questionnaire; GD = gender distribution; M = males; n = number; NA = not applicable; NR = not reported; NSD = no significant differences; P = parallel; R = randomized; RYGB = Roux-en-Y gastric bypass; SS = statistically significant; *v.* = versus; VAS = visual analogue scale; vol = volume; wt = weight.

[a] Low stress was induced by giving the participants an easy Sudoku puzzle; high stress was induced by giving the participants an impossible Sudoku puzzle.

[b] Levels of inherent sugar in the juice were not provided.
